# Optimizing sparse sequencing of single cells for highly multiplex copy number profiling

**DOI:** 10.1101/gr.188060.114

**Published:** 2015-05

**Authors:** Timour Baslan, Jude Kendall, Brian Ward, Hilary Cox, Anthony Leotta, Linda Rodgers, Michael Riggs, Sean D'Italia, Guoli Sun, Mao Yong, Kristy Miskimen, Hannah Gilmore, Michael Saborowski, Nevenka Dimitrova, Alexander Krasnitz, Lyndsay Harris, Michael Wigler, James Hicks

**Affiliations:** 1 Cold Spring Harbor Laboratory, Cold Spring Harbor, New York 11724, USA;; 2 Department of Molecular and Cellular Biology, Stony Brook University, Stony Brook, New York 11790, USA;; 3 Sigma-Aldrich Research Technology, Saint Louis, Missouri 63103, USA;; 4 Phillips Research North America, Biomedical Informatics, Briarcliff Manor, New York 10510, USA;; 5 Division of Hematology/Oncology, Department of Medicine, Case Western Reserve School of Medicine, Cleveland, Ohio 44106, USA;; 6 Department of Pathology, University Hospitals Case Medical Center and Case Western Reserve University, Cleveland, Ohio 44106, USA;; 7 Clinic for Gastroenterology, Hepatology, and Endocrinology, Hannover Medical School, 30625 Hannover, Germany;; 8 Seidman Cancer Center, University Hospitals of Case Western, Cleveland, Ohio 44106, USA

## Abstract

Genome-wide analysis at the level of single cells has recently emerged as a powerful tool to dissect genome heterogeneity in cancer, neurobiology, and development. To be truly transformative, single-cell approaches must affordably accommodate large numbers of single cells. This is feasible in the case of copy number variation (CNV), because CNV determination requires only sparse sequence coverage. We have used a combination of bioinformatic and molecular approaches to optimize single-cell DNA amplification and library preparation for highly multiplexed sequencing, yielding a method that can produce genome-wide CNV profiles of up to a hundred individual cells on a single lane of an Illumina HiSeq instrument. We apply the method to human cancer cell lines and biopsied cancer tissue, thereby illustrating its efficiency, reproducibility, and power to reveal underlying genetic heterogeneity and clonal phylogeny. The capacity of the method to facilitate the rapid profiling of hundreds to thousands of single-cell genomes represents a key step in making single-cell profiling an easily accessible tool for studying cell lineage.

Tumor cells evolve via the acquisition of somatic genetic lesions that bestow the capacity to proliferate and survive (Vogelstein et al. 2013). Consequently, genetically distinct subpopulations are likely to evolve and dynamically interact with each other (Marusyk et al. 2012; Yates and Campbell 2012; Burrell et al. 2013). The presence of tumor genome heterogeneity has long been acknowledged (Nowell 1976), and recent investigations have tied it to disease progression and metastasis, as well as therapeutic resistance (Turke et al. 2010; Walter et al. 2012; Wu et al. 2012). Unfortunately, our knowledge of cancer genome heterogeneity is still lacking, due primarily to the lack of sensitive approaches that explore genetic heterogeneity at a genome-wide scale. New technologies are needed to facilitate the dissection of intra-tumoral heterogeneity.

Recently, with the advent of next-generation sequencing (NGS) technologies and whole-genome amplification (WGA) approaches, single-cell genomic investigations have emerged as a powerful approach to analyze cancer genetic heterogeneity (Navin et al. 2011; Baslan et al. 2012). Genome-wide single-cell sequencing investigations have begun to illuminate valuable and novel aspects of cancer biology and promise to deliver more (Ni et al. 2013; Dago et al. 2014; Francis et al. 2014; Lohr et al. 2014). To realize the potential of single-cell sequencing in understanding the biology of heterogeneity, methods are needed that allow the investigation of hundreds of single-cell genomes at a reasonable cost in time, effort, and reagents. Sequencing hundreds of single cells to the nucleotide level is simply not affordable even with the remarkable NGS platforms that are available. Fortunately, copy number analysis requires only sparse sequence coverage, yet it can distinguish subpopulations and provides deep insights into genetic heterogeneity. Thus, in theory, coupling sparse sequencing with molecular barcoding approaches offers a means to profile many cells together.

Indeed, we and others have recently demonstrated the feasibility of this approach by combining up to eight barcoded single cells on a single sequencing lane (McConnell et al. 2013; Dago et al. 2014), but the potential for higher level multiplexing has not been explored at either the bioinformatic or operational levels. To accomplish this, informatic analysis aimed at identifying minimal sequence read requirements for robust copy number identification is required. Furthermore, while technically feasible, amplifying and creating barcoded sequencing libraries from many single cells using traditional library preparation protocols involving sonication, end repair, A-tailing, and adaptor ligation is time-consuming and expensive. We have therefore set out to create an optimized multiplexing process by determining the minimum number of reads that can be used to determine genome-wide copy number profiles at specific levels of resolution and then to develop a simplified preparative method that is faster and cheaper and yet maximizes the amount of information that can be extracted from each sequencing read from a single sequencing lane of the Illumina HiSeq machine.

Here, we describe a robust and affordable, high-throughput method that employs a modified version of degenerate oligonucleotide priming-PCR (DOP-PCR) amplification, simplified library preparation, and multiplex sequencing that facilitates the retrieval of the genome-wide copy number landscape of hundreds of individual cancer cells. Our method drastically lowers the cost of profiling single-cell genomes (down to ∼$30 per single cell), significantly cuts sequence library preparation time, and maximizes the amount of information extracted from each single-cell sequencing data set. We apply our approach to human cancer cell lines and clinical cancer biopsies to demonstrate its power to reveal population heterogeneity.

## Results

### Optimizing coverage in a multiplexing strategy

**Figure 1.**
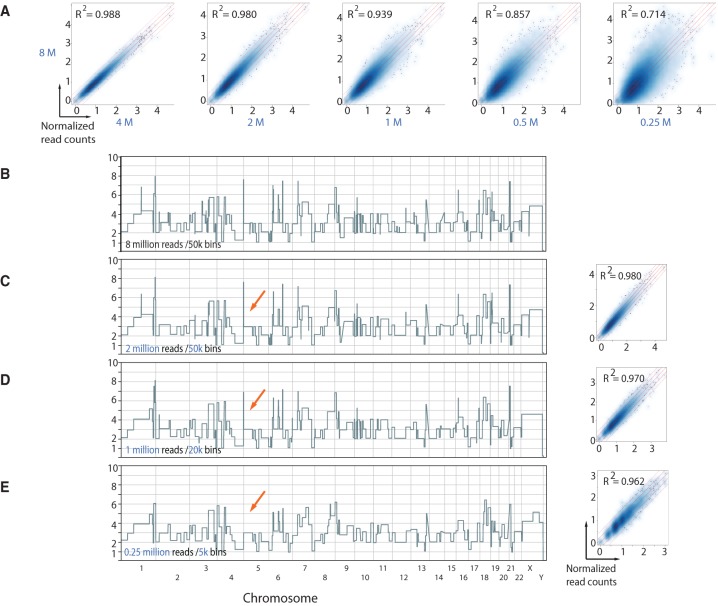
Down-sampling analysis reveals minimal data requirements for copy number determination. (*A*) Density scatter correlation plots of the normalized bin read counts (directly proportional to copy number) of the original 8 million uniquely mapped read data set with data sets down-sampled to 4, 2, 1, 0.5, and 0.25 million reads using 50K bins. (*B*) Genome-wide CNV plot of rearranged cancer cell with 8 million reads using 50K bins. (C) Genome-wide CNV view of same cell with data sets down-sampled to 2 million reads using 50K bins. Box plot illustrates normalized read count scatter correlation plots with original 8 million read data set. (*D*,*E*) Same as in *C* but with 1 million and 0.25 million reads using 20K and 5K bins, respectively. Red arrows exemplify CNVs that are lost with decreasing resolution (i.e., fewer number of bins). Pearson's correlation coefficients of data sets are displayed.

CNV analysis by sequencing typically counts the number of reads that uniquely map to bioinformatically computed segments or “bins” of genomic sequence (Alkan et al. 2009; Chiang et al. 2009). We have recently shown, from sequencing data of uniformly amplified single-cell genomic DNA, that the copy number of a particular bin is directly proportional to the number of sequencing reads that map within it (Navin et al. 2011; Baslan et al. 2012). We used 50,000 bins (50K bins), with an average bin length of 60 kb. The profiles produced have clean breakpoints and segments with quantal values, as one expects from single-cell data. At the published coverage, this averaged 160 maps per bin, clearly an excess. But how much data (measured as the number of sequencing reads per bin) is required to produce a clean, quantal, genome-wide copy number profile from a single cell at 50K bin resolution? Although the answer can be approached mathematically on assumptions about binomial sampling distributions, the confident detection of minimum features, and expectations of quantal values, we decided to take an empirical approach using the same cancer cells previously analyzed. We retrieved single-cell sequencing data (Navin et al. 2011) for a rearranged cancer cell (DNA content = 2.95N) for which 8 million uniquely mapped reads were available and performed correlation and copy number analysis on down-sampled data sets. Normalized read counts of data down-sampled to 4, 2, 1, 0.5, and 0.25 million reads plotted against the original 8 million reads data set demonstrate strong correlations down to 1 million reads (*R*^2^ = 0.939) (Fig. 1A). The 2 million read copy number profile (∼40 reads per bin) was highly similar to the profile generated from the original 8 million read single-cell data set using 50K bins (Fig. 1B,C). Using fewer reads than this retained features of the breakpoint profile, but the quantal nature of the copy number segments became less clear (Supplemental Fig. S1). Two million uniquely mapped reads were also sufficient to recapitulate the copy number landscape of tumor cells with different DNA content (Supplemental Fig. S2). Thus, irrespective of DNA content, 2 million uniquely mapped reads are sufficient to retrieve the genome-wide copy number profile of a single cell when dividing the genome into 50K bins.

Are 50K bins needed? Given that the majority of copy number alterations found in bulk analysis of tumor genomes are on the order of megabases (Mb) or greater (Beroukhim et al. 2010), we reasoned that decreasing the number of bins (i.e., increasing bin lengths) would decrease sequencing read requirements for copy number determination. Reanalyzing the down-sampled data using 20K and 5K bins (calculated using the variable bin method) (see Methods) revealed that strong correlations were maintained with the original 8 million data set down to 1 million and 0.25 million uniquely mapped reads for 20K and 5K bins, respectively (Fig. 1D,E; Supplemental Fig. S3). Importantly 96% and 75% of the breakpoints, detected at a resolution of 50K bins, were called at bin resolutions of 20K and 5K, respectively, with the down-sampled data (Fig. 1D,E). Moreover, the quantal nature of the copy number segments is clearly maintained. Naturally, at lower resolutions of 20K and 5K bins, some focal alterations were missed (Fig. 1D,E, red arrows). One and 0.25 million sequencing reads for 20K and 5K bins, respectively, were also sufficient to retrieve genome-wide CNV information in cancer cells with different DNA content (Supplemental Fig. S2).

We extended the down-sampling analysis to four single cells with DNA content of 2.95N (aneuploid), three cells with DNA content of 1.6N (hypodiploid) as well as four apparently normal diploid cells. All cells were down-sampled to 2 million, 1 million, and 0.25 million and analyzed using 50K, 20K, and 5K bins, respectively. For each cell and sample size, 100 downsamples were compared to a sample of 6 million reads. The diploid cells had identical copy number calls for 99% of the genome for all sample sizes. The hypodiploid cell downsamples matched the copy number calls for the 6 million read samples 99%, 92%, and 90%, respectively, for 2 million, 1 million, and 250,000 read samples. The downsamples for the aneuploid cells matched 93%, 84%, and 78%. The bin-to-bin differences are mainly due to slight changes in boundaries of segments (Fig. 1; Supplemental Fig. S4). The data together, when taking into account current average HiSeq output of 200 million reads per lane, indicate that, theoretically, up to 500 single-cell genomes can be multiplexed and analyzed on a single HiSeq lane. Table 1 lists the multiplexing capacity and the genomic bin resolution given different bin sizes.

**Table 1.**
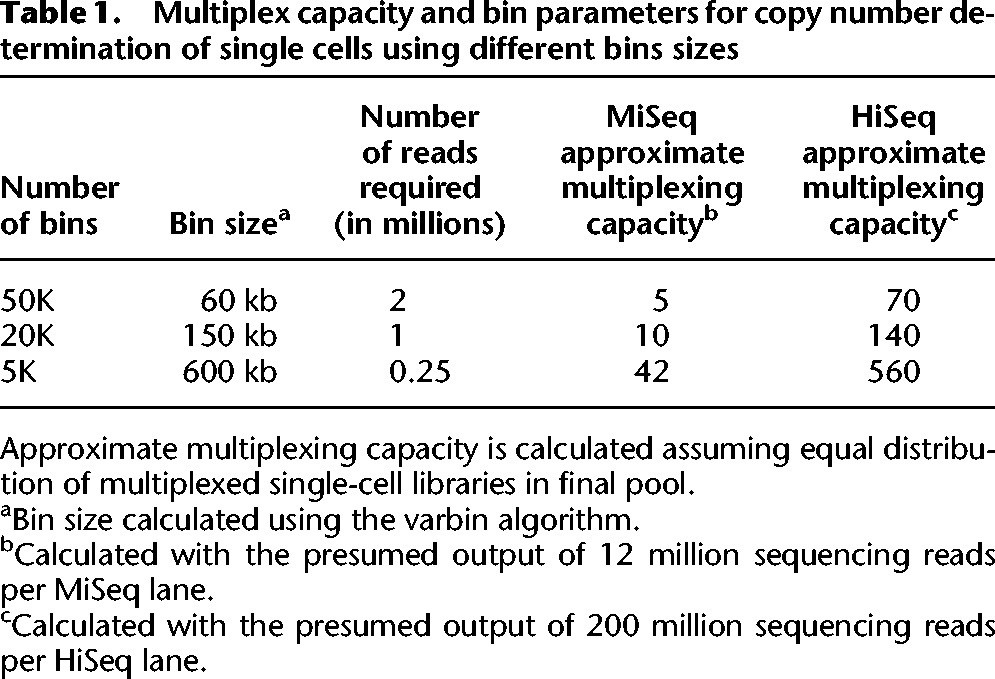
Multiplex capacity and bin parameters for copy number determination of single cells using different bins sizes

### An optimized DOP-PCR molecular approach for high-level multiplexing

We employ DOP-PCR methodology for WGA because it amplifies more uniformly across the genome than other methods, and when the goal is CNV analysis, we obtain more reproducible results with lower noise (Navin et al. 2011; Baslan et al. 2012; Cai et al. 2014).

Maximizing the efficiency of sequencing by identifying minimal read requirements to facilitate multiplexing is not the only problem that needs to be addressed to optimize the efficiency of highly multiplex single-cell CNV profiling. Performing the steps of WGA and library preparation protocols, involving sonication, end repair, A-tailing, and ligation for each cell individually takes a great deal of bench work and can cost as much as $50 per cell in reagents alone, making the procedure itself a target for optimization. Moreover, the resulting DNA molecules following DOP-PCR carry universal 30-bp sequences at the ends and even when sonicated, the universal DOP primer sequences remain on a substantial fraction of the DNA molecules, causing decreased complexity (Supplemental Fig. S5), lower quality data, and decreased mappability for some reads.

**Figure 2.**
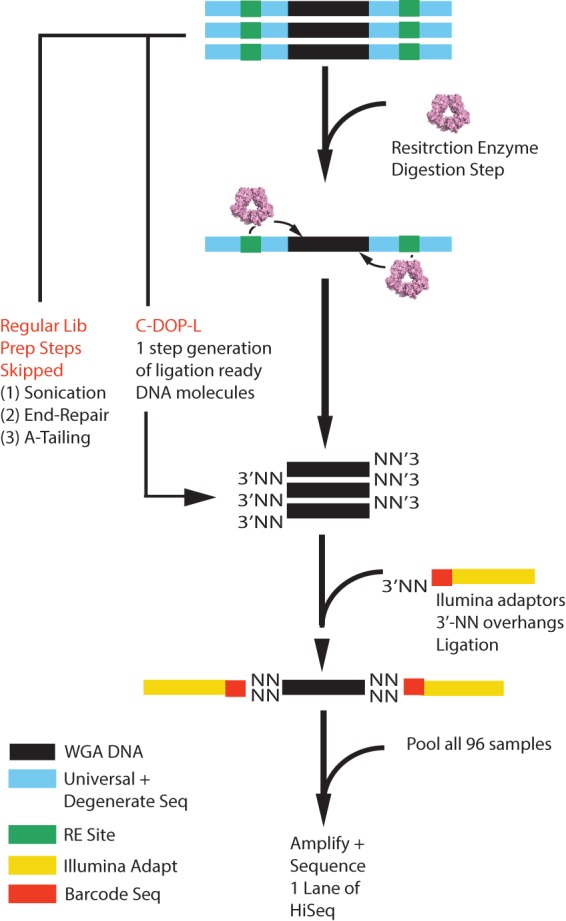
Schematic overview of the C-DOP-L approach for highly multiplex single-cell sequencing. In brief, WGA DNA is treated with restriction enzyme to cleave the universal sequences found at the ends of WGA DNA. The digestion reaction leaves 3′-NN overhangs (where N is any base: A,T,C,G). Digested DNA is then ligated to barcoded Illumina sequencing adaptors that are designed to contain 3′-NN overhangs. After barcode addition, samples are pooled, amplified, and sequenced on a single lane of the HiSeq instrument. (WGA) Whole-genome amplified, (RE) restriction enzyme, (N) any base (A,T,C, or G).

To circumvent the above-mentioned issues, we devised a method, termed Cleavable DOP-PCR Ligation (C-DOP-L), which incorporates restriction enzyme digestion of the universal sequences at the ends of WGA DNA via the SEQXE kit (Sigma-Aldrich), with an “NN-mediated” DNA ligation of barcoded Illumina adaptors (Fig. 2). In our method, single-cell genomes are amplified using DOP-PCR similar to what we have reported before (Navin et al. 2011; Baslan et al. 2012). However, the degenerate oligonucleotide differs in that it incorporates a recognition site for a type IIS restriction enzyme (isoschizomers AcuI and Eco57I [CTGAAG 16/14]). When added to the WGA DNA, the enzyme recognizes its binding site and cleaves 16/14 (top/bottom strand) bases away from its recognition sequence, effectively removing the entire universal sequence found at the ends of the DNA molecules. Furthermore and importantly, the digestion leaves 3′-NN overhangs (where N is any base). These overhangs are subsequently used in the ligation of barcoded Illumina adaptors designed to carry 3′-NN overhangs on the P5 adaptor. To test the method, we designed and synthesized 96 modified Illumina adaptors carrying custom barcoded adaptors with sufficient complexity (equal distributions of A, T, C, and G base pairs) in the first 4 bases (Supplemental Fig. S6).

### Validation of C-DOP-L with cell lines

To ensure that the modification of the degenerate oligonucleotide primer does not affect the uniformity of the WGA reaction or introduce distortions to the genome, we examined normal and cancer cell lines. We chose to examine ∼100 genomes per HiSeq Illumina lane, a convenient number for microplate processing, and aimed for ∼1.5 million reads per cell.

**Figure 3.**
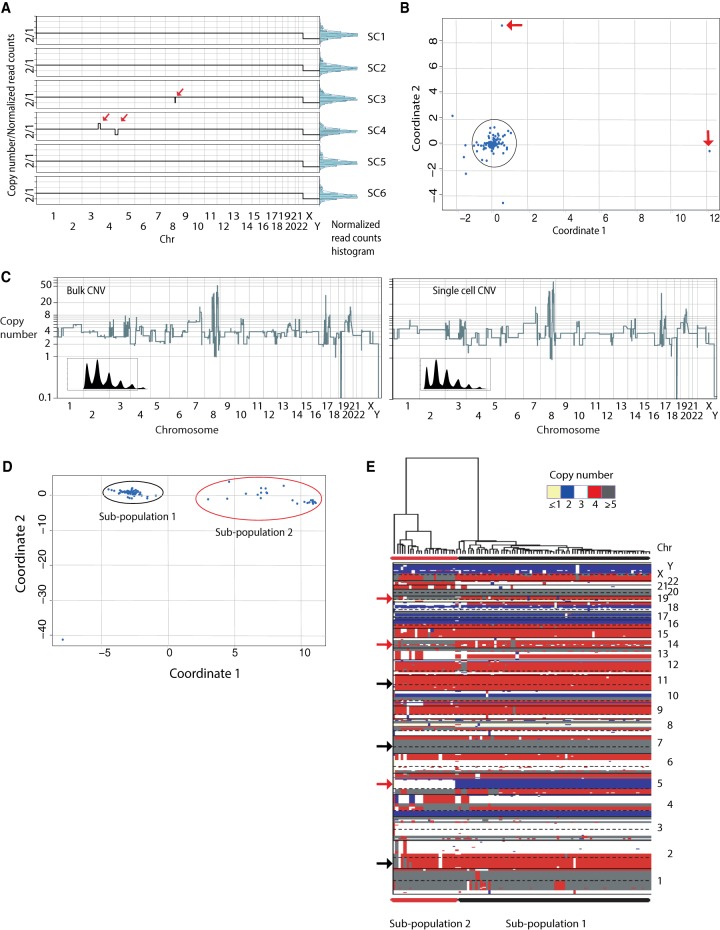
C-DOP-L approach provides uniform, unbiased amplification of single-cell genomes, accurate determination of copy number states and reveals genomic heterogeneity in breast cancer cell lines. (*A*) Genome-wide copy number plots of single cells at 5K bins of a diploid karyotypically normal lymphoblastoid cell line from a male (315A) and histogram distributions of normalized read count data. Red arrows point to examples of somatically mosaic integer copy number events that are observed in some single cells. Data plotted are of nonrounded copy number estimates. (*B*) Multi-dimensional scaling of 95 single cells from 315A. Red arrows point to outlier cells. (*C*) Copy number profile of a single SK-BR-3 cell (*right*) compared to bulk from millions of cells (*left*) at 5K bins. Boxed inserts display smoothened kernel density plots of normalized read counts showing discrete densities (quantal data). (*D*) Multidimensional scaling of 94 single SK-BR-3 cells. (*E*) Hierarchical clustering copy number heatmap of single-cell SK-BR-3 genomes. Black arrows denote copy number alterations shared by the vast majority of single cells. Red arrows denote copy number alterations that distinguish the two subpopulations.

We began by flow sorting nuclei from a diploid EBV immortalized lymphoblastoid cell line (315A) derived from a normal male, selecting for diploid nuclei, making 96 single depositions, and amplifying each. Of the 96 sorted single nuclei, 95 were successfully amplified (i.e., yielding a minimum of 2 µg of total WGA DNA), processed using the C-DOP-L method, and sequenced on a single lane of HiSeq 2000. Sequencing reads for single cells were de-convoluted, mapped to the human genome, and processed using our variable bin algorithm for copy number determination (Navin et al. 2011; Baslan et al. 2012; see Methods). For all the single cells processed, we obtained on average 1.5 million uniquely mapped reads with a range of 0.25 to 3.6 million with all cells having a minimum of 0.25 million reads (Supplemental Fig. S7A). Sequenced single-cell DNA displayed a GC amplification bias that was comparable in magnitude to our previous approach and was easily corrected using lowess smoothing (Supplemental Fig. S7B). Importantly, the C-DOP-L method maintained the minimal sequence bias exhibited in our previous work using the DOP-PCR approach (Navin et al. 2011; Baslan et al. 2012; Cai et al. 2014). The uniformity of the amplification reaction was maintained, as demonstrated by the tight histogram distributions of the normalized read count data as well as the genome-wide copy number profiles, revealing the vast majority of the genome at copy number 2 (Fig. 3A; Supplemental Fig. S7C). In aggregate, 0.97% of bins across the 95 cells have a copy number differing from two on the autosomes and one on the sex chromosomes. This represents an upper bound for false-positive copy number calls. The false-negative rate was assessed by randomly inserting 1000 segments of lengths five, nine, and 13 bins representing copy numbers 1 and 3 in randomly selected autosomes across all 95 cells. Segments were considered false-negative if the copy number of the central bin was called incorrectly. The false-negative rates for copy number 1 were 12%, 1%, and 0% for segment sizes five, nine, and 13 bins, respectively. The corresponding false-negative rates for copy number 3 were 27%, 6%, and 1%.

Multidimensional scaling of the 315A single-cell copy number profiles showed tight clustering for the majority of single cells (88 single cells out of 96 sequenced single cells) (Fig. 3B). All of these 88 cells displayed consistent normal genome-wide copy number profiles with all of the autosomes at copy number 2 and the sex chromosomes at copy number 1, attesting to the reproducibility of the method (Fig. 3A). Two cells were distant in the multidimensional scaling graph from the cluster (Fig. 3B, red arrows) with one cell displaying a chromosome wide duplication of Chromosome 2 and another cell displaying heterozygous focal deletions on multiple chromosomes (Supplemental Fig. 8A,B). Another five cells (outside of the black circle in Fig. 3B) displayed deviations from discrete integer copy number profiles and more spread distributions of normalized read count data (Supplemental Fig. S8C,D,E). These profiles could be the result of an error in the WGA amplification process, or cells caught early in S phase of the cell cycle. We occasionally observe the occurrence of nonrecurrent focal deletions or duplications (Fig. 3A, red arrows) in otherwise normal cells. The nature of these events is currently unknown and likely represents somatic events.

To further validate our approach, we proceeded to profile single nuclei from a rearranged human breast cancer cell line. Flow sorting 96 single nuclei from the pseudo-triploid (apparent DNA content 3.65N by FACS) breast cancer cell line SK-BR-3 followed by WGA amplification and C-DOP-L library preparation resulted in 94 successfully amplified and ligated products (97.9%). These were loaded on a single HiSeq 2000 lane and, after informatic processing, produced genome-wide copy number profiles (20K bins) that very closely recapitulated that of the corresponding SK-BR-3 bulk DNA (*R*^2^ Pearson's correlation = 0.963) (Fig. 3C). Importantly, smoothing kernel density plots of the normalized sequencing data revealed the quantized nature of the single-cell data with densities corresponding to discrete copy number integer values (Fig. 3C, insert boxes; Supplemental Fig. S7D). In addition, presumed driver genomic alterations observed in the bulk copy number profile, such as high level amplification of the *MYC* locus on Chromosome 8, the heterozygous deletion of *DCC* on Chromosome 18, and the homozygous deletion of a cluster of zinc finger proteins on Chromosome 19, were observed in 100% of the single cells sequenced (Supplemental Fig. S9). Interestingly, multidimensional scaling of all 94 integer copy number profiles resolved two distinct clusters corresponding to a major subpopulation (subpopulation 1) and a minor subpopulation (subpopulation 2) (Fig. 3D). Hierarchal clustering of the single-cell profiles plotted in the form of a copy number heatmap clearly illustrates that the two subpopulations are derived from the same lineage, with the vast majority of the genome present at the same copy number in almost all single cells, for example, Chromosomes 2, 7, and 11 (Fig. 3E, black arrows). Importantly, the two subpopulations differed significantly with different copy number states on Chromosomes 5, 14, and 19, among others (Fig. 3E, red arrows; Supplemental Fig. S10A). Some of these events are also evident in the bulk SK-BR-3 copy number profile as segments with noninteger copy number values (Supplemental Fig. S10B). This genomic heterogeneity of a cancer cell line is not restricted to SK-BR-3, as another breast cancer cell line (MDA-MB-231) also revealed substantial heterogeneity where three distinct subpopulations were observed (Supplemental Fig. S11). Thus, the data demonstrate the robustness and accuracy of our highly multiplex single-cell sequencing approach in profiling cancer genomic heterogeneity.

### Highly multiplex single-cell sequencing of clinical breast cancer tissue reveals subclonal populations and somatic mosaicism of chromosomal amplifications

**Figure 4.**
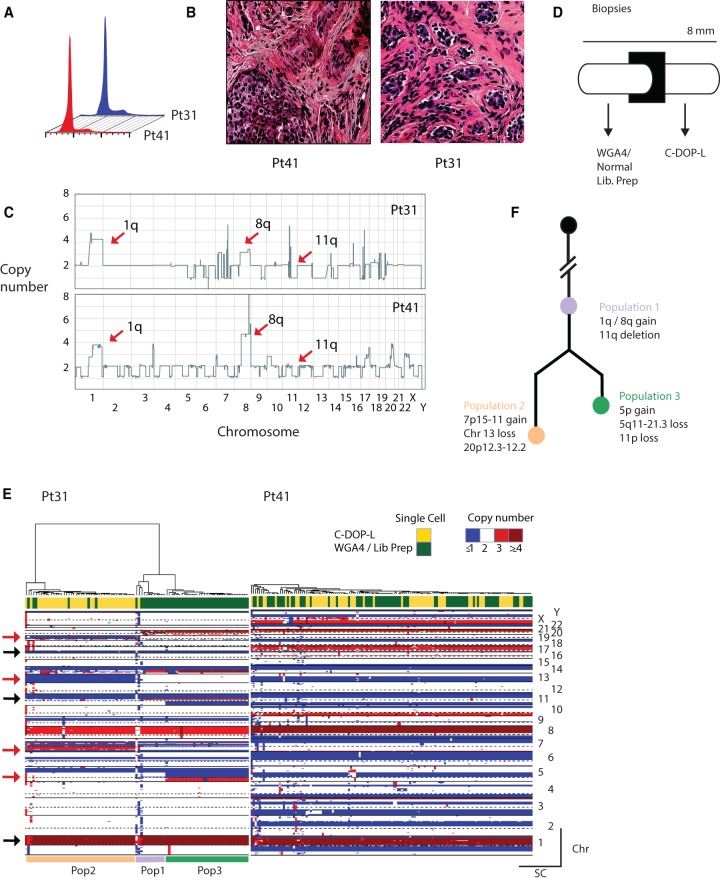
Highly multiplex single-cell sequencing identifies genomic heterogeneity in breast cancer biopsies and informs evolutionary history. (*A*) Flow cytometric profiles of the clinical cases. (*B*) Similar histopathologies of the biopsies showing invasive ductal carcinoma with moderate differentiation and complex glandular growth pattern. (*C*) Bulk genome-wide copy number profile of the two cases. Arrows point to genomic regions recurrently gained or lost in ER + breast cancer. (*D*) Schematic representation of biopsy sectioning and processing using single-cell methods. (*E*) Hierarchical clustering heatmap of the Pt41 and Pt31. Arrows point to genomic regions shared by both tumor genomes (black) and genomic regions that display heterogeneity in Pt31 (red). (*F*) Evolutionary history of Pt31 subpopulations.

To determine the feasibility of high-level multiplexing for actual clinical samples, we analyzed two estrogen receptor (ER)-positive breast cancer cases (Pt31 and Pt41) from a larger study in progress. Both were determined to be diploid in DNA content, with similar histopathology and from the same gene expression subtype (luminal B) as determined by RNA sequencing and PAM50 analysis (Fig. 4A,B; Supplemental Fig. S12). Bulk copy number analysis revealed characteristic ER-positive copy number alterations, such as gains of Chromosomes 1q and 8q and deletion of Chromosome 11q (Fig. 4C; Russness et al. 2010; Curtis et al. 2012) in both cases. To allow comparison with our previous approach (WGA4 amplification [Sigma-Aldrich] followed by standard Illumina library prep.), core needle biopsies from both cases (8 mm in length) were cut evenly into two sections for processing using WGA4 and C-DOP-L (Fig. 4D). For each section, 96 nuclei were sorted, and the plates were processed with either WGA4 or C-DOP-L. Each 96 multiplexed pool was sequenced on a single lane of the Illumina HiSeq instrument. Cells yielding at least 0.25 million uniquely mapped reads were considered successful for the complete process. Compared with the cell lines, the clinical samples were somewhat more variable. The number of successfully profiled cells for Pt41 was 86/96 using WGA4 and 89/96 using C-DOP-L, while Pt31 yielded 88/96 and 69/96, respectively. The homogeneity of the single-cell copy number profiles from Pt41 (discussed more below) allowed us to compare differences in data quality between the two methods. Using a method based on a median absolute pairwise difference metric (MAPD) (Cai et al. 2014) to compare the two methods shows the normalized bin count data to be somewhat noisier with C-DOP-L, with a MAPD of 0.30 compared to 0.25 for WGA4. However, comparing copy number, the two methods are almost identical, with a median pairwise copy number difference of 3.4% for WGA4 and 3.9% for C-DOP-L.

Single tumor cells from both cases were then plotted and clustered in a heatmap format based on their genome-wide copy number profile (Fig. 4E). We omitted from the figure the cells with normal profiles and used CORE (Cores of Recurrent Events) (Krasnitz et al. 2013; see Methods) to select the cancer cells that are part of a clonal lineage. We were thus able to approximate tumor cellularity for each biopsy (∼60% tumor for Pt31 and ∼90% tumor for Pt41). Chromosome 1q and 8q duplications as well as the loss of 11q were found in virtually all single cells from both tumors using both approaches, consistent with these events occurring very early in the evolution of the tumor genome and further attesting to the sensitivity and specificity of our approach (Fig. 4E, black arrows). Interestingly, whereas the Pt41 tumor profile contained more copy number alterations than Pt31 (measured as % of genome altered), single-cell copy number profiles from Pt41 displayed homogeneity, with almost all cells sharing all chromosomal alterations. In contrast, Pt31 had three subpopulations that differed in their copy number status at multiple chromosomes, for example Chromosomes 5, 7, 11, and 13 (Fig. 4E, red arrows). These populations were also found to differ in proportion between the two adjacent sections. Phylogenetic analysis of the subpopulations based on their genomic alterations revealed that the two divergent populations, 2 and 3, arose from the earlier ancestral population 1 via the acquisition of additional genomic alterations (Fig. 4F), yet, interestingly, we see that population 1 has persisted.

**Figure 5.**
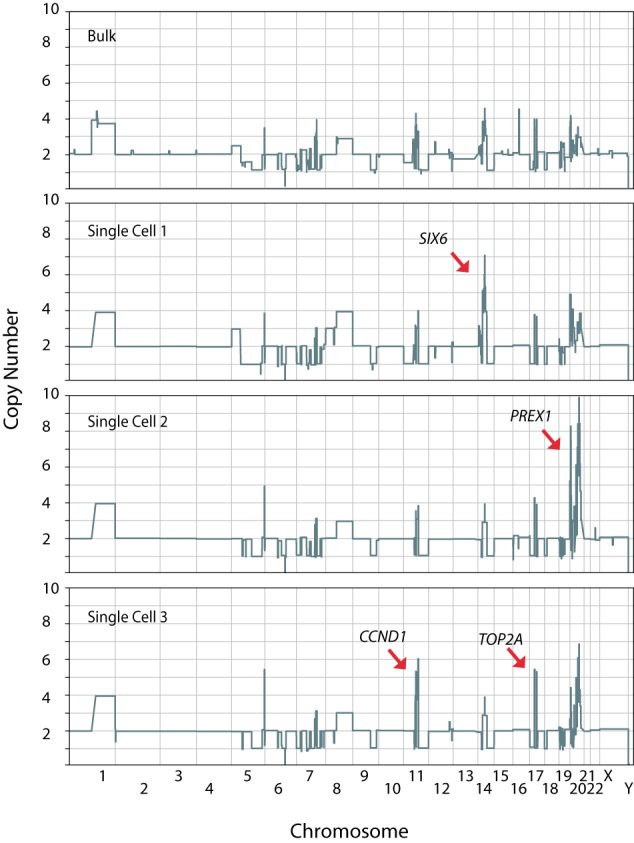
Single-cell copy number analysis reveals somatic mosaicism of chromosomal amplifications in Pt31. Genome-wide CNV plots at 20K bin resolution of representative single cells containing mosaic amplicons compared to bulk. (*SIX6*) SIX homeobox 6, (*PREX1*) phosphatidylinositol-3,4,5-triphosphate-dependent Rac exchange factor 1, (*CCND1*) cyclin D1, (*TOP2A*) topoisomerase (DNA) II alpha.

Upon further examination of the single-cell copy number profiles of the tumor (Pt31), we noticed additional heterogeneity: mosaic copy number amplification variants (Fig. 5). Some occurred at genes with established clinical significance in breast cancer, such as the amplification of cyclin D1 (*CCND1*) (Arnold and Papanikolaou 2005) on Chromosome 11 and topoisomerase (DNA) II alpha (*TOP2A*) (Engstrom et al. 2014) on Chromosome 17, while others occurred at genes for which experimental evidence exists for involvement in cancer, such as the homeobox protein *SIX6* (Soulier et al. 2005) on Chromosome 14 and *PREX1* (Sosa et al. 2010) on Chromosome 20. Together, these data provide strong evidence of the power of highly multiplex single-cell sequencing in resolving subclonal structure and illustrating genomic heterogeneity present within the genomes of human tumors.

## Discussion

The potential of single-cell genome profiling in understanding cancer heterogeneity lies in the ability to profile hundreds and even thousands of single-cell genomes. Our approach extends the burgeoning field of single-cell genomics by offering a robust high-throughput method to examine the genome-wide copy number profile of hundreds of single cancer cells. Our down-sampling simulation analysis facilitated the benchmarking of the minimal data requirements necessary to reproduce genome-wide copy number variation of cancer cells and guided our subsequent multiplexing strategy. By coupling the restriction enzyme digestion of the WGA DNA universal sequences with NN-mediated adaptor ligation, our approach allows us to (1) maximize the amount of information extracted from each sequencing read via the elimination of the WGA universal sequences, (2) enhance the quality of the sequencing output, and (3) significantly reduce the cost and effort required to generate highly multiplexed single-cell sequencing libraries. In our previous report (Navin et al. 2011), each single cell was sequenced on a single lane of the Illumina platform at a cost of ∼1000. Using the methods described here, with the multiplexing of 96 single cells on a single HiSeq lane, we have reduced the cost of sequencing a single cell to ∼30 per cell in reagents and sequencing costs (refer to Supplemental Table S1 for breakdown of the method's cost and time effort). Undoubtedly, with the decreasing cost per base from NGS, this figure is likely to drop even further and facilitate the profiling of thousands of single cells in a single lane. At that stage, microfluidics will be needed to reduce preparation costs and manual labor. Also, our approach using C-DOP-L can easily accommodate different multiplexing platforms such as the Illumina third read TruSeq indexing system.

While our approach focuses on robustly identifying an important class of somatic mutations in copy number variants, it does not focus on the identification of other sources of somatic mutations such as single nucleotide variants (SNVs) and structural variants. However, it is important to point out that, with the current sequencing output of NGS platforms, it is still prohibitively expensive to sequence the exomes of hundreds of single cells. Furthermore, even though our single-cell amplification approach (degenerate oligonucleotide priming-PCR) does not cover the entire genome when sequenced at high depths, there is evidence to suggest that up to a third of the whole genome can be covered in a single-cell WGA product (Voet et al. 2013), and we observe that genome coverage increases with more single cells sequenced (Supplemental Fig. S13). Thus, an approach based on initially resolving clonal population structure via genome-wide copy number variation followed by pooling of single-cell libraries and targeted capture of particular subpopulations (for example, the three subpopulations in Pt31) may provide exome-wide views of these subpopulations. A similar strategy has recently proven effective in illustrating the clonal architecture of secondary acute myeloid leukemia (Hughes et al. 2014).

Importantly, the robustness of our approach has allowed us to profile hundreds of single-cell genomes from cancer cell lines and human tissue, and the resulting data have provided unique biological insights with important implications for tumor biology. First, the observation of subclonal variation in human cancer cell lines, generally presumed to be monoclonal, implies that the evolutionary process that underlies cancer development is still operative in cell culture. Second, subclonal heterogeneity in culture raises the question of how similar cancer cell lines are between different laboratories and how to compare different studies utilizing the same cell lines (Hatzis et al. 2014). Third, the observation of the relative homogeneity of Pt41 in comparison to Pt31 is intriguing given that the Pt41 genome is more highly rearranged. This might suggest that factors other than genomic instability might modulate intra-tumoral heterogeneity and/or that diversification is dynamic throughout the history of a tumor. And fourth, the mosaicism of genomic amplifications observed in Pt31 highlights the remarkable heterogeneity cancer genomes are capable of sustaining and begs the question of how these varied alterations might modulate responses in the face of selective pressures such as therapeutic intervention.

Finally, while we devised our method for the purpose of studying cancer heterogeneity and evolution, it is clear that its applications are not limited to cancer biology (Baslan and Hicks 2014). The robustness of the method coupled with its high-throughput nature makes it an attractive approach to examine the CNV patterns underlying aneuploidy in human gametes (Hou et al. 2013) as well as human neurons (McConnell et al. 2013). In addition, biological phenomena such as the ploidy conveyor in hepatocytes (Duncan et al. 2010) could very well be carefully dissected using the methods described here. With regard to cancer biology, the application of our high-throughput single-cell genome sequencing approach to many tumor types and ultimately hundreds of cancer samples is bound to illuminate the underlying biology behind tumor heterogeneity and help in our struggle to better understand and tackle this disease.

## Methods

### Sequence read down-sampling analysis

Down-sampling analysis was performed on 11 cells from Navin et al. (2011), where each single cell had a minimum of 6 million mapped reads after removing duplicates. The 11 cells consisted of four diploid cells (SRR053620, SRR053623, SRR053624, SRR053633), three hypodiploid cells (SRR054569, SRR089401, SRR089402), and four aneuploid cells (SRR054609, SRR054610, SRR054611, SRR054612). Sample sizes of 2 million, 1 million, and 250,000 were sampled 100 times each for each cell and compared to a 6 million read sample. Reads were binned and data segmented to obtain copy number estimates across the genome. The 6 million read and 2 million read samples were binned using 50,000 bins. The 1 million read samples were binned using 20,000 bins. The 250,000 read samples were binned using 5000 bins. Each of the bins in the 50,000 bin genome partition (small bins) was matched to a bin in the 20,000 and 5000 bin partitions (large bins) by selecting the large bin that most overlapped the small bin. Copy number calls in the 6 million read samples were compared to the copy number calls in the corresponding bins in the other downsamples.

### Nuclei isolation from cell cultures and clinical samples, DNA staining, and single-cell flow cytometry

315A lymphoblastoid cells were cultured as suspension cultures in RPMI 1640 (Gibco-Invitrogen) supplemented with 10% FBS (HyClone), 100 units mL^−1^ penicillin, and 100 μg mL^−1^ streptomycin (Gibco-Invitrogen). SK-BR-3 and MDA-MB-231 were cultured as adherent cultures in DMEM (Gibco-Invitrogen) supplemented with 10% FBS, 100 units mL^−1^ penicillin, and 100 μg mL^−1^ streptomycin. All lines were cultured at 37°C and 5% CO_2_. Core biopsies, obtained prior to treatment, were processed by formalin fixation and paraffin embedding (FFPE) or frozen down and stored in OCT compound (two cores each per biopsy event). Both specimen types were subjected to sectioning, hematoxylin and eosin staining, and histologic evaluation by the study pathologist. Frozen cores were processed for single-nuclei isolation as described before (Baslan et al. 2012). FPPE sections were used for tumor histology and immunohistochemistry. For cell lines (both adherent and suspension), nuclei were prepared by collecting ∼10^6^ cells in a 15-mL conical centrifuge tube and gently centrifuging at 105*g* for 4 min followed by medium aspiration and the addition of 1 mL of NST-DAPI buffer. Nuclei were prepared from frozen core biopsy samples by finely mincing tissue in 0.5 mL NST-DAPI buffer according to a protocol previously published by Baslan et al. (2012). Single-cell sorting was performed using a FACS AriaIIU SORP (BD Biosciences) with the ACDU option (Automated Cell Deposition Unit). The sorter was run inside a BioProtect IV Safety Cabinet (Baker Company) to maintain BSL2 biosafety standards. The DAPI signal was detected by a 355-nM UV laser (450/50 bandpass filter). Gains were set for the UV photomultiplier based on the DNA content equivalent to human diploid lymphoblast cells. Single nuclei were determined by doublet discrimination as described by Wersto et al. (2001). Single cells were deposited in a 96-well plate format containing 9 µL of cell lysis buffer (800 µL H_2_O, 6 µL Proteinase K, and 96 µL 10× singe cell lysis and fragmentation buffer [Sigma WGA4]).

### Whole-genome amplification and Illumina library generation

Single cells were lysed by incubating 96-well plates for 1 h at 50°C, followed by 4 min at 99°C using a thermocycler. Single-cell whole-genome amplification was then carried out using the SeqPlex Enhanced DNA Amplification Kit (SEQXE, Sigma) according to the manufacturer's instructions. Twenty-four amplification cycles were used. Single-cell amplification products were purified using QIAquick 96-well plates according to the manufacturer's instructions. DNA was eluted in 50 µL EB solution. All subsequent reactions were carried out in 96-well plate format using multichannel pipetting. Restriction digestion of WGA universal sequences was performed interchangeably using SeqPlex supplied Primer Removal reagents (Sigma) and Eco57I (Thermo Scientific). One microgram of WGA DNA products in a total volume of 20 µL containing 2.4 µL 10× Primer Removal Buffer/Buffer G, 0.4 µL Primer Removal Solution/SAM, and 0.5 Primer Removal Enzyme/Eco57I enzyme (Thermo Scientific). Reactions were incubated at 37°C for 30 min followed by incubation at 65°C for 15 min for enzyme deactivation. Reactions were subsequently cooled on ice. Following restriction digestion, 24 µL of EB and 26 µL of 2× Quick Ligase Reaction Buffer (NEB) were added to each reaction to bring the volume up to 70 µL. The addition of 26 µL of 2× Quick Ligase Reaction Buffer is critical since it facilitates selection of higher molecular weight DNA (between 200 and 600 bps). Digested DNA was subsequently purified using Agencourt AMPure XP beads (Beckman Coulter) according to the following protocol: 30 µL of warmed beads were added to each digestion reaction. Beads and reaction products were mixed by vortexing for 7 sec. Mixed reactions were then incubated off-magnet for 10 min at RT, after which they were then transferred to a DynaMag-96 Side magnet (Life Technologies) and left to stand for 5 min. Ninety microliters of supernatant were withdrawn and discarded. Beads were washed with 180 µL of freshly made 80% EtOH. After a second round of EtOH washing, beads were allowed to dry on the magnet for 15 min. Dried beads were then resuspended off-magnet in 48 µL of EB and allowed to incubate for 10 min, followed by 5 min incubation on-magnet. Forty-four microliters of the elutant were then mixed with 26 µL of 2× Quick Ligase Reaction Buffer and purified again using AMPure XP beads according to the steps described above. The final elution volume was 44 µL of EB, of which 41 µL were transferred to another 96-well plate for ligation. HPLC-purified barcoded NN-Illumina adaptors were ordered from IDT. Two microliters of barcoded adaptors (PE5/7) were added to each bead-purified, digested WGA DNA. Ligation reactions were carried in a total volume of 70 µL with 1 µL of ligase and 26 µL of ligase buffer. Reactions were incubated at 20°C for 30 min followed by a DNA ligase inactivation step at 65°C for 15 min. Heat-inactivated ligation reactions were subsequently cooled at 4°C. After adaptor ligation, 2.3 µL of each 96 adaptor ligated library were pooled together and distributed equally into three fresh tubes (∼70 µL). Pools were purified 1× using 30 µL beads as described above and eluted in 30 µL of buffer EB. Following bead purification of the pools, PCR enrichment was performed in a total volume of 62.5 µL containing 2.5 µL of 10 µM PE5/7 primers and 30 µL of Phusion High-Fidelity PCR Master Mix (NEB) according to the following parameters: (1) 98°C for 30 sec, (2) 98°C for 10 sec, (3) 65°C for 30 sec, and (4) 72°C for 30 sec, (5) return to (2) for a total of 10 cycles, then (6) 72°C for 5 min, and (7) hold at 4°C. Samples were then quantified using the Bioanalyzer and qPCR and subsequently run on HiSeq machines.

### Variable bin (varbin) method

In dividing the genome into bins for copy number estimation, we utilize a method that partitions the genome into bins of variable sizes based on the unique mappability of sequences across the human genome, with each bin containing the same number of mappable positions. Bin boundaries were computed for 50K, 20K, and 5K bins according to the guidelines outlined by Baslan et al. (2012). Additionally, for a number of regions in the genome, we noticed the accumulation of very high read depth compared to the expected norm. These regions we found to consistently display the high read depth in both bulk as well as single-cell sequencing data, and many were found in bins surrounding centromeres. Using data from 54 normal diploid single cells, these bins (designated as “bad bins”) were determined as follows. Bincounts were divided by the mean for each cell to normalize for differences in total read count between each cell. For each chromosome, the mean of the bins over all cells is subsequently subtracted from each normalized bin count to normalize for differences between chromosomes. The mean and standard deviation of the autosomes was then used to compute an outlier threshold corresponding to a *P*-value of 1/*N*, where *N* is the number of bins used. This was done for the 5K, 20K, and 50K bin data sets. These bins are masked from downstream copy number analysis.

### Sequence alignment and single-cell copy number analysis

Multiplexed single-cell sequencing libraries were split according to their unique barcode identifiers specified by the first seven bases of the sequencing reads. Single-cell sequencing data were aligned to the human reference genome hg19 using Bowtie (Langmead et al. 2009). Reads were sorted, PCR duplicates removed, and then indexed using SAMtools (Li et al. 2009). Uniquely mapping reads were counted for each bin and normalized for GC bias using lowess smoothing. Normalized read count data were then segmented using circular binary segmentation (CBS) (Venkatraman and Olshen 2007). For copy number estimation in single cells, we employed an approach based on least-squares fit as follows: When analyzing data from a single cell, the copy number at any point in the genome must be an integer. Thus, if the data were accurate, then after segmentation, the segmented mean values should have a clear multimodal distribution with a peak representing each copy number present in the genome. The data at this point in the analysis are centered around 1, meaning that the mean value across the bins (5000, 20,000, or 50,000) of the segmented value is close to 1. In a diploid genome, this would represent a copy number of 2, with regions of copy number 1 having a segmented value near 0.5 and regions of copy number 3 having a segmented value near 1.5. These could easily be converted to copy number estimates by multiplying the segmented value for each bin by 2 and rounding to the nearest integer. This is the basic idea used to estimate copy number in single-cell data. In rearranged cancer cells where the copy number of genomic segments is unknown, in order to find the best multiplier we multiply the segmented profile by 1.5, 1.55, 1.6, 1.65, … 5.5 (81 different values) and compute what we call a quantal error for each multiplier. This is the sum of the squared difference between the multiplied segmented profile and the multiplied segmented profile rounded to the nearest integer. The multiplier that gives the smallest quantal error is deemed the best fit and used to estimate copy number. This quantal error can also be used as a quality control parameter. Cells with a large quantal error can really be multiple cells, parts of cells, or have degraded DNA. For heatmap plots, single cells were hierarchically clustered based on their genome-wide copy number profiles using the Manhattan distance function and clustered according to the Ward method.

### False-negative estimation

The false-negative rate was estimated by randomly inserting simulated segments of copy numbers 1 and 3 into randomly selected cells from the set of 95 cells from the 315A cell line. Segments of 5, 9, and 13 bins were simulated by picking random contiguous bins within the autosomes that did not overlap centromeres or chromosome boundaries. To simulate copy number 1, the normalized read count was multiplied by 0.5 and the standard deviation from 0.5 increased by √2. To simulated copy number 3, the normalized read count was multiplied by 1.5 and the standard deviation from 1.5 decreased by √1.5. The genome with the inserted segment was then segmented using DNAcopy and considered matched if the copy number call at the central bin in the segment matched the exacted copy number; otherwise, the segment was counted as a false negative.

### Consistency

To compare data quality between the WGA4 and the C-DOP-L methods, we use an approach based on the median absolute pairwise difference (MAPD) quality control metric (Cai et al. 2014). Tumor cells from sample Pt41 appear to be from the same clone for cells processed with both WGA4 and C-DOP-L, allowing a direct comparison of methods. To estimate noise in the bin ratio data between two cells, we used the median absolute deviation (MAD) of the difference in bin ratio data between the two cells. This is the pairwise distance. For any given cell, the median of the pairwise distances between that cell and the other cells processed with the same protocol is the MAPD for that cell. We report the median MAPD for the cells from both methods. To estimate variation in copy number calls, we count the number of bins with different copy number calls comparing pairs of cells. The MAPD for each cell is the median number of differences compared to the other cells processed by the same protocol. We report the median of these MAPD values for both protocols as a percentage of the total number of bins.

### DNA purification of bulk samples and Illumina library generation

For bulk extraction of genomic DNA from cell lines as well as clinical tissue, leftover nuclei suspensions were subjected to phenol-chloroform DNA extraction (details are provided in Supplemental Material). Purified DNA was processed using standard Illumina library preparation methods and sequenced on the HiSeq instrument. Sequencing data was analyzed for copy number variation using the same methods described for single-cell analysis (above).

### RNA purification, RNA-seq library generation, and analysis

RNA was purified from homogenized cancer biopsy samples, and RNA sequencing libraries were prepared using the Ovation RNA-seq system (NuGEN). Sequence data were processed using a variety of algorithmic tools including Mapsplice2 (Wang et al. 2010) and RSEM (RNA-seq by Expectation Maximization) (Li et al. 2010). Detailed information is included in the Supplemental Material.

### CORE

CORE analysis was performed as described before (Krasnitz et al. 2013). Briefly, segments with integer copy number values above or below the reference were considered amplified or deleted, respectively. Copy number events in each cell were derived by slicing, and cores, i.e., regions of significantly recurrent (*P* < 0.05) gains and losses, were determined by applying the CORE method to the entire set of single-cell genomes. Finally, the incidence table was computed, with rows and columns corresponding, respectively, to cells and cores and with values in the [0,1] interval quantifying the best match between an event in the cell and the core. Single cells that contained statistically significant cores we judged to be part of the cancer phylogeny and used for downstream analysis, while cells lacking cores (mostly cells with the vast majority of the genome at copy number 2) we judged to be contaminating normal cells.

## Data access

The sequencing data from this study have been submitted to the NCBI Sequence Read Archive (SRA; http://www.ncbi.nlm.nih.gov/sra) under accession number SRP055057.

## Competing interest statement

Brian Ward is an employee of Sigma-Aldrich. Mao Yong and Nevenka Dimitrova are employees of Phillips Research North America.

## Supplementary Material

Supplemental Material

## References

[BASLANGR188060C2] Alkan C, Kidd JM, Marques-Bonet T, Aksay G, Antonacci F, Hormozdiari F, Kitzman JO, Baker C, Malig M, Mutlu O, 2009 Personalized copy number and segmental duplication maps using next-generation sequencing. Nat Genet 41: 1061–1067. 19718026 10.1038/ng.437 PMC2875196

[BASLANGR188060C3] Arnold A, Papanikolaou A. 2005 Cyclin D1 in breast cancer pathogenesis. J Clin Oncol 23: 4215–4224. 15961768 10.1200/JCO.2005.05.064

[BASLANGR188060C4] Baslan T, Hicks J. 2014 Single cell sequencing approaches for complex biological systems. Curr Opin Genet Dev 26C: 59–65. 25016438 10.1016/j.gde.2014.06.004

[BASLANGR188060C5] Baslan T, Kendall J, Rodgers L, Cox H, Riggs M, Stepansky A, Troge J, Ravi K, Esposito D, Lakshmi B, 2012 Genome-wide copy number analysis of single cells. Nat Protoc 7: 1024–1041. 22555242 10.1038/nprot.2012.039 PMC5069701

[BASLANGR188060C6] Beroukhim R, Mermel CH, Porter D, Wei G, Raychaudhuri S, Donovan J, Barretina J, Boehm JS, Dobson J, Urashima M, 2010 The landscape of somatic copy-number alterations across human cancers. Nature 463: 899–905. 20164920 10.1038/nature08822 PMC2826709

[BASLANGR188060C7] Burrell RA, McGranahan N, Bartek J, Swanton C. 2013 The causes and consequences of genetic heterogeneity in cancer evolution. Nature 501: 338–345. 24048066 10.1038/nature12625

[BASLANGR188060C8] Cai X, Evrony GD, Lehmann HS, Elhosary PC, Mehta BK, Poduri A, Walsh CA. 2014 Single-cell, genome-wide sequencing identifies somatic copy-number variation in the human brain. Cell Rep 5: 1280–1289. 25159146 10.1016/j.celrep.2014.07.043 PMC4272008

[BASLANGR188060C10] Chiang DY, Getz G, Jaffe DB, O'Kelly MJ, Zhao X, Carter SL, Russ C, Nusbaum C, Meyerson M, Lander ES. 2009 High-resolution mapping of copy-number alterations with massively parallel sequencing. Nat Methods 6: 99–103. 19043412 10.1038/nmeth.1276 PMC2630795

[BASLANGR188060C11] Curtis C, Shah SP, Chin SF, Turashvilli G, Rueda OM, Dunning MJ, Speed D, Lynch AG, Samarajiwa S, Yaun Y, 2012 The genomic and transcriptomic architecture of 2,000 breast tumours reveals novel subgroups. Nature 486: 346–352. 22522925 10.1038/nature10983 PMC3440846

[BASLANGR188060C12] Dago AE, Stepansky A, Carlsoon A, Luttgen M, Kendall J, Baslan T, Kolatkar A, Wigler M, Bethel K, Gross ME, 2014 Rapid phenotypic and genomic change in response to therapeutic pressure in prostate cancer inferred by high content analysis of single circulating tumor cells. PLoS One 9: e101777. 25084170 10.1371/journal.pone.0101777 PMC4118839

[BASLANGR188060C13] Duncan AW, Taylor MH, Hickey RD, Hahlon Newell AE, Lenzi ML, Olson SB, Finegold MJ, Grompe M. 2010 The ploidy-conveyor of mature hepatocytes as a source of genetic variation. Nature 467: 707–710. 20861837 10.1038/nature09414 PMC2967727

[BASLANGR188060C14] Engstrom MJ, Ytterhus B, Vatten LJ, Opdahl S, Bofin AM. 2014 TOP2A gene copy number change in breast cancer. J Clin Pathol 67: 420–425. 24403186 10.1136/jclinpath-2013-202052 PMC3995265

[BASLANGR188060C15] Francis JM, Zhang CZ, Maire CL, Jung J, Manzo VE, Adalsteinsson VA, Homer H, Haidar S, Blumenstiel B, Padamallu CS, 2014 EFGR variant heterogeneity in glioblastoma resolved through single-nucleus sequencing. Cancer Discov 4: 956–971. 24893890 10.1158/2159-8290.CD-13-0879 PMC4125473

[BASLANGR188060C16] Hatzis C, Bedard PL, Birkbak NJ, Beck AH, Aerts HJ, Stem DF, Shi L, Clarke R, Quackenbush J, Haibe-Kains B. 2014 Enhancing reproducibility in cancer drug screening: how do we move forward? Cancer Res 74: 4016–4023. 25015668 10.1158/0008-5472.CAN-14-0725 PMC4119520

[BASLANGR188060C17] Hou Y, Fan W, Yan L, Li R, Lian Y, Huang J, Li J, Xu L, Tang F, Xie XS, 2013 Genome analysis of single human oocytes. Cell 155: 1492–1506. 24360273 10.1016/j.cell.2013.11.040

[BASLANGR188060C18] Hughes AE, Magrini V, Demeter R, Miller CA, Fulton R, Fulton LL, Eades WC, Elliot K, Heath S, Westervelt P, 2014 Clonal architecture of secondary acute myeloid leukemia defined by single-cell sequencing. PLoS Genet 10: e1004462. 25010716 10.1371/journal.pgen.1004462 PMC4091781

[BASLANGR188060C20] Krasnitz A, Sun G, Andrews P, Wigler M. 2013 Target inference from collections of genomic intervals. Proc Natl Acad Sci 110: E2271–E2278. 23744040 10.1073/pnas.1306909110 PMC3690846

[BASLANGR188060C21] Langmead B, Trapnell C, Pop M, Salzberg SL. 2009 Ultrafast and memory-efficient alignment of short DNA sequences to the human genome. Genome Biol 10: 2078–2079. 10.1186/gb-2009-10-3-r25 PMC2690996 19261174

[BASLANGR188060C22] Li H, Handsaker B, Wysoker A, Fennell T, Ruan J, Homer N, Marth G, Abecasis G, Durbin R; 1000 Genome Project Data Processing Subgroups. 2009 The Sequence Alignment/Map format and SAMtools. Bioinformatics 15: 2078–2079. 19505943 10.1093/bioinformatics/btp352 PMC2723002

[BASLANGR188060C23] Li B, Routti V, Stwart RM, Thomson JA, Dewey CN. 2010 RNA-Seq gene expression estimation with read mapping uncertainty. Bioinformatics 26: 493–500. 20022975 10.1093/bioinformatics/btp692 PMC2820677

[BASLANGR188060C24] Lohr JG, Adalsteinsson VA, Cibulskis K, Choudhury AD, Rosenberg M, Cruz-Gordillo P, Francis JM, Zhang CZ, Shalek AK, Satija R, 2014 Whole-exome sequencing of circulating tumor cells provides a window into metastatic prostate cancer. Nat Biotechnol 32: 479–484. 24752078 10.1038/nbt.2892 PMC4034575

[BASLANGR188060C25] Marusyk A, Almendro V, Polyak K. 2012 Intra-tumor heterogeneity: a looking glass for cancer? Nat Rev Cancer 12: 323–334. 22513401 10.1038/nrc3261

[BASLANGR188060C26] McConnell MJ, Lindberg MR, Brennard KJ, Piper JC, Voet T, Cowing-Zitron C, Shumilina S, Lasken RS, Vermeesch JR, Hall IM, 2013 Mosaic copy number variation in human neurons. Science 342: 632–637. 24179226 10.1126/science.1243472 PMC3975283

[BASLANGR188060C27] Navin N, Kendall J, Troge J, Andrews P, Rodgers L, McIndoo J, Cook K, Stepansky A, Levy D, Esposito D, 2011 Tumour evolution inferred by single-cell sequencing. Nature 472: 90–94. 21399628 10.1038/nature09807 PMC4504184

[BASLANGR188060C28] Ni X, Zhuo M, Su Z, Duan J, Gao Y, Wang Z, Zong C, Bai H, Chapman AR, Zhao J, 2013 Reproducible copy number variation patterns among single circulating tumor cells of lung cancer patients. Proc Natl Acad Sci 110: 21083–21088. 24324171 10.1073/pnas.1320659110 PMC3876226

[BASLANGR188060C29] Nowell PC. 1976 The clonal evolution of tumor cell populations. Science 194: 23–28. 959840 10.1126/science.959840

[BASLANGR188060C31] Russness HG, Vollan HK, Lingjaerde OC, Krasnitz A, Lundin P, Naume B, Sorlie T, Borgen E, Rye IH, Langerod A, 2010 Genome architecture characterizes tumor progression paths and fate in breast cancer patients. Sci Transl Med 2: 38ra47. 10.1126/scitranslmed.3000611 PMC3972440 20592421

[BASLANGR188060C32] Sosa MS, Lopez-Haber C, Yang C, Wang H, Lemmon MA, Busillo JM, Luo J, Benovic JL, Klein-Szanto A, Yagi H, 2010 Identification of the Rac-GEF P-Rex1 as an essential mediator of ErbB signaling in breast cancer. Mol Cell 40: 877–892. 21172654 10.1016/j.molcel.2010.11.029 PMC3038344

[BASLANGR188060C33] Soulier J, Clappier E, Cayuela JM, Regnault A, Garcia-Peydro M, Dombert H, Baruchel A, Toribio ML, Sigaux F. 2005 HOXA genes are included in genetic and biologic networks defining human acute T-cell leukemia (T-ALL). Blood 106: 274–286. 15774621 10.1182/blood-2004-10-3900

[BASLANGR188060C34] Turke AB, Zejnullahu K, Wu YL, Song Y, Dias-Santagata D, Lifshits E, Toschi L, Rogers A, Mok T, Sequist L, 2010 Preexistence and clonal selection of MET amplification in EGFR mutation NSCLC. Cancer Cell 17: 77–88. 20129249 10.1016/j.ccr.2009.11.022 PMC2980857

[BASLANGR188060C35] Venkatraman ES, Olshen AB. 2007 A faster circular binary segmentation algorithm for the analysis of array CGH data. Bioinformatics 23: 657–663. 17234643 10.1093/bioinformatics/btl646

[BASLANGR188060C36] Voet T, Kumar P, Van Loo P, Cooke SL, Marshall J, Lin ML, Zamani Esteki M, Van der Aa N, Mateiu L, McBride DJ, 2013 Single-cell paired-end genome sequencing reveals structural variation per cell cycle. Nucleic Acid Res 41: 6119–6138. 23630320 10.1093/nar/gkt345 PMC3695511

[BASLANGR188060C37] Vogelstein B, Papadopoulos N, Velculescu VE, Zhou S, Diaz LA Jr, Kinzler KW. 2013 Cancer genome landscapes. Science 339: 1546–1558. 23539594 10.1126/science.1235122 PMC3749880

[BASLANGR188060C38] Walter MJ, Shen D, Ding L, Shao J, Koboldt DC, Chen K, Larson DE, McLellan MD, Dooling D, Abbott R, 2012 Clonal architecture of secondary acute myeloid leukemia. N Engl J Med 366: 1090–1098. 22417201 10.1056/NEJMoa1106968 PMC3320218

[BASLANGR188060C39] Wang K, Singh D, Zeng Z, Coleman SJ, Huang Y, Savich GL, He X, Mieczowski P, Grimm SA, Perou CM, 2010 MapSplice: accurate mapping of RNA-seq reads for splice junction discovery. Nucleic Acids Res 38: e178. 20802226 10.1093/nar/gkq622 PMC2952873

[BASLANGR188060C40] Wersto RP, Chrest FJ, Leary JF, Morris C, Stetler-Stevenson MA, Gabrielson E. 2001 Doublet discrimination in DNA cell-cycle analysis. Cytometry 46: 296–306. 11746105 10.1002/cyto.1171

[BASLANGR188060C41] Wu X, Northcott PA, Dubuc A, Dupuy AJ, Shih DJ, Witt H, Croul S, Bouffet E, Fults DW, Eberhart CG, 2012 Clonal selection drives genetic divergence of metastatic medulloblastoma. Nature 482: 529–533. 22343890 10.1038/nature10825 PMC3288636

[BASLANGR188060C42] Yates LR, Campbell PJ. 2012 Evolution of the cancer genome. Nat Rev Genet 137: 795–806. 23044827 10.1038/nrg3317 PMC3666082

