# Supplementary material for: Optimizing sparse sequencing of single cells for highly multiplex copy number profiling
Source: Genome Res. 2015 May;25(5):714–24. doi: 10.1101/gr.188060.114 (PMC4417119; doi:10.1101/gr.188060.114)
Supplement: Supplemental Material [file supp_25_5_714__index.html]

Optimizing sparse sequencing of single cells for highly multiplex copy number profiling — Optimizing sparse sequencing of single cells for highly multiplex copy number profiling — Supplemental Material 

# Optimizing sparse sequencing of single cells for highly multiplex copy number profiling

## Supplemental Material

**Files in this Data Supplement:**

- Supplemental Material.pdf
