# Supplementary material for: Optimizing sparse sequencing of single cells for highly multiplex copy number profiling
Source: Genome Res. 2015 May;25(5):714–24. doi: 10.1101/gr.188060.114 (PMC4417119; doi:10.1101/gr.188060.114)
Supplement: Supplemental Material [file supp_gr.188060.114_Supplemental_Material.pdf]

SUPPLEMENTARY MATERIAL

Optimizing sparse sequencing of single cells for highly multiplex copy number profiling

Timour Baslan[1,2,9], Jude Kendall[1], Brian Ward[3], Hilary Cox[1], Anthony Leotta[1], Linda Rodgers[1], Michael Riggs[1], Sean D'Italia[1], Guoli Sun[1], Mao Yong[4], Kristy Miskimen[5], Hannah Gilmore[6], Michael Saborowski[7] Nevenka Dimitrova[4], Alexander Krasnitz[1], Lyndsay Harris[5,8], Michael Wigler[1], and James Hicks[1,10].

1. Cold Spring Harbor Laboratory (CSHL), Cold Spring Harbor, New York, 11724.
2. Department of Molecular and Cellular Biology (MCB), Stony Brook University, Stony Brook, New York, 11790.
3. Sigma-Aldrich Research Technology, Saint Louis, Missouri, 63103.
4. Phillips Research North America, Biomedical Informatics, Briarcliff Manor, NY, 10510.
5. Division of Hematology/Oncology, Department of Medicine, Case Western Reserve School of Medicine, Cleveland, Ohio, 44106.
6. Department of Pathology, University Hospitals Case Medical Center and Case Western Reserve University, Cleveland, OH, 44106.
7. Clinic for Gastroenterology, Hepatology, and Endocrinology, Hannover Medical School, Hannover, Germany, 30625.
8. Seidman Cancer Center, University Hospitals of Case Western, Cleveland, 44106.
9. Current address: Department of Cancer Biology and Genetics, Memorial Sloan Kettering Cancer Center, New York, 10065.
10. To whom correspondence should be addressed: hicks@cshl.edu

CONTENTS

1. SUPPLEMENTARY METHODS

1.1 DNA purification of bulk samples and Illumina library generation.
1.2 RNA purification, RNA-Seq library generation and analysis.

2. SUPPLEMENTARY REFERENCES

3. SUPPLEMENTARY FIGURES

3.1 Supplementary Figure 1: Down-sampling beyond 2 million reads using 50K bins results in loss of quantal nature of the CNV data.
3.2 Supplementary Figure 2: Identified minimal read requirement parameters apply to rearranged cancer cells that display varying DNA content.
3.3 Supplementary Figure 3: Correlation plots of down-sampled data sets from single cell in Figure 1 across different bin lengths.
3.4 Supplementary Figure 4: Downsampling analysis of copy number variation in single cell genomes of varying ploidy levels at different bin resolutions.
3.5 Supplementary Figure 5: C-DOP-L approach removes WGA sequenced to restore nucleotide diversity in sequenced DNA.
3.6 Supplementary Figure 6: Schematic representation of the sequences of the custom Illumina barcodes utilized in the C-DOP-L method.
3.7 Supplementary Figure 7: C-DOP-L approach facilitates robust determination of high quality CNV data from single cells.
3.8 Supplementary Figure 8: Genome alterations found in normal cells.
3.9 Supplementary Figure 9: Potential "driver" copy number alterations in the SK-BR-3 genome are identified in 100% of single cancer cells sequenced.
3.10 Supplementary Figure 10: Copy number variation between cells of the different SK-BR-3 sub-populations.
3.11 Supplementary Figure 11: Intra-tumoral heterogeneity and sub-clonal populations in the MDA-MB-231 breast cancer cell line.
3.12 Supplementary Figure 12: Pt41 and Pt31 belong to the Luminal B breast cancer gene expression subgroup.

3.13 Supplementary Figure 13: Genome coverage increased with more single cells sequenced

4. SUPPLEMENTARY TABLE

4.1 Supplementary Table 1: Cost and time estimates of the C-DOP-L method

SUPPLEMENTARY METHODS

DNA purification of bulk samples and Illumina library generation

For bulk extraction of genomic DNA from cell lines as well as clinical tissue, leftover nuclei suspensions (from which single cells were retrieved) were mixed with equal volume of 2X lysis buffer (1 ml 1M Tris-HCl pH 8.0, 200 µl 0.5M EDTA pH 8.0, 200 µl 5M NaCl, 500 µl 10% SDS, 1ml 1M DTT, 1.1ml H2O). Lysis nuclei mixtures were then treated with 50 µl Proteinase K (20mg/ml) and incubated for 16 hrs at 55 °C. Digestion mixture was allowed to cool to room temperature followed by RNase A treatment using 5 µl of 20mg/ml RNase A. RNase A treatment was performed at 37 °C for 1 hr. Genomic DNA was then purified from Proteinase K and RNase A treated nuclei using phenol-chloroform extraction as follows: Equal volume of Phenol was added to nuclei digestion mixtures and allowed to mix gently in a rotator for 10 min. Mixtures were then spun at 13,000g at 4 °C. Aqueous phase material was carefully retrieved (avoiding interface material) and saved in a fresh tube. Phenol extraction was repeated 2X. Phenol extracted material was further purified by adding equal volume of Phenol:Chloroform:Isoamyl alcohol. Mixing, centrifugation and aqeous phase extraction were performed as described above. Phenol:Chrloroform:Isoamyl alcohol extraction was repeated 1X. DNA was then further extracted using Chloroform:Isoamyl alcohol following the steps as described above. Chloroform extraction was also repeated 1X. Chloroform extracted DNA was subsequently precipitated by adding 1/10 volume of 3M NaOAc pH 5.2, with gentle mixing, followed by the addition of equal volume Isopropanol, mixing by inverting the tube ~40X and centifigation at 13,000g for 30 min at 4 °C. Supernatent was removed by pipetting carefully as not to disturb the pellet. DNA pellets were washed with 300 µl cold 70% EtOH (1X) followed by an additional wash using 300 µl cold 100% EtOH (1X). DNA pellets were allowed to dry at room temp for ~ 15 min and re-suspended in H2O at 4 °C overnight. 0.25 – 1 ug of high molecular weight genome DNA was then sonicated using the Covaris machine at 300+/- using the following parameters: duty cycle – 10%, Intensity – 4, cycles/burst – 200, and time 80 s. Sonicated genomic DNA was then prepared for Illumina library generation using In-house custom built barcoded adaptors previously described (Iossifov et al. 2012), with the exception that bead purification was performed 2X.

RNA purification, RNA-Seq library generation and analysis

Core biopsies were removed from the OCT and homogenized in lysis buffer using Hard Tissue Omni Tip Homogenizing Probes. DNA and RNA were extracted from the lysate using the Qiagen AllPrep DNA/RNA Mini Kit (Qiagen). RNA concentrations and 260/280 ratios were determined using a NanoDrop. RNA integrity was assessed using a Bioanalyzer (Agilent). Fifty to 100 ng of total RNA were reverse-transcribed and amplified using the Ovation RNA-Seq System (NuGEN). Amplified cDNA was purified using the Qiagen MinElute Reaction Cleanup Kit (Qiagen) and quantified using a NanoDrop. RNA-Sequencing was performed on the amplified cDNA at the Yale Center for Genome Analysis (West Haven, CT) or Expression Analysis, Inc. (Durham, NC). Paired-end sequencing was performed on the Illumina GAII platform using amplified total RNA with 74bp read length, yielding data on transcript abundance for a total of 22,160 genes and 34,449 transcripts, yielding about 50M reads per sample. Raw sequencing data were analyzed using RNA-SEQ Version 2 pipeline. Reads were aligned to human reference genome hg19 with Mapsplice2 (Wang et al. 2010) and gene expression was quantitated using RSEM (RNA-SEQ by Expectation Maximization) (Li et al. 2010). Each gene expression profile (Pt31 and Pt41) was normalized in the same manner as the TCGA breast cancer cohort, by setting the upper quartile value to 1000. To perform subtyping, a nearest centroid classifier was implemented using TCGA level-3 gene expression profiles along with study samples. Out of 1100 TCGA breast cancer (BRCA) samples with available gene expression data, 542 samples were mapped out with subtype information from the published study (TCGA et al. 2012), including 96 Basal-like, 58 Her2-Enriched, 231 LumA, 128 LumB and 29 Normal-like samples. With the log2 transformed gene expression data aligned on PAM50 list (Parker et al. 2009), centroids for samples from each subtype were estimated and further used to subtype BrUOG samples based on 'nearest distance' criteria. Pt31 and Pt41 gene expression profiles were pre-processed in the same manner prior to the prediction. For visualization purpose, a principal component analysis was conducted to

identify the top principal components (PCs) based on pre-processed 542 TCGA breast cancer samples. Using the top 2 principal components (PC), the samples were projected onto the PC subspace. In the PCA analysis, the top 2 principal components were capable in explaining 37.7% and 25.6% of the variance of the TCGA sample matrix respectively.

SUPPLEMENTARY REFERENCES

Iossifov I, Ronemus , Levy D, Wang Z, Hakker I, Rosenbaum J, Yamrom B, Lee YH, Narzisi G, Leotta A, et al. 2012. De novo gene disruptions in children on the autistic spectrum. *Neuron* **74:** 285-299.

Li B, Routti V, Stwart RM, Thomson JA, Dewey CN. 2010. RNA-Seq gene expression estimation with read mapping uncertainty. *Bioinformatics* **26:** 493-500.

Parker JS, Mullins M, Cheang MC, Leung S, Voduc D, Vickery T, Davies S, Fauron C, He X, Hu Z, et al. 2009. Supervised risk predictor of breast cancer based on intrinsic subtypes. *J Clin Oncol* **27:** 1160-1167.

The Cancer Genome Atlas Network. 2012. Comprehensive molecular portraits of human breast tumours. *Nature* **490:** 61–70.

Wang K, Singh D, Zeng Z, Coleman SJ, Huang Y, Savich GL, He X, Mieczowski P, Grimm SA, Perou CM, et al. 2010. MapSplice: accurate mapping of RNA-seq reads for splice junction discovery. *Nucleic Acids Res* **38:** e178.

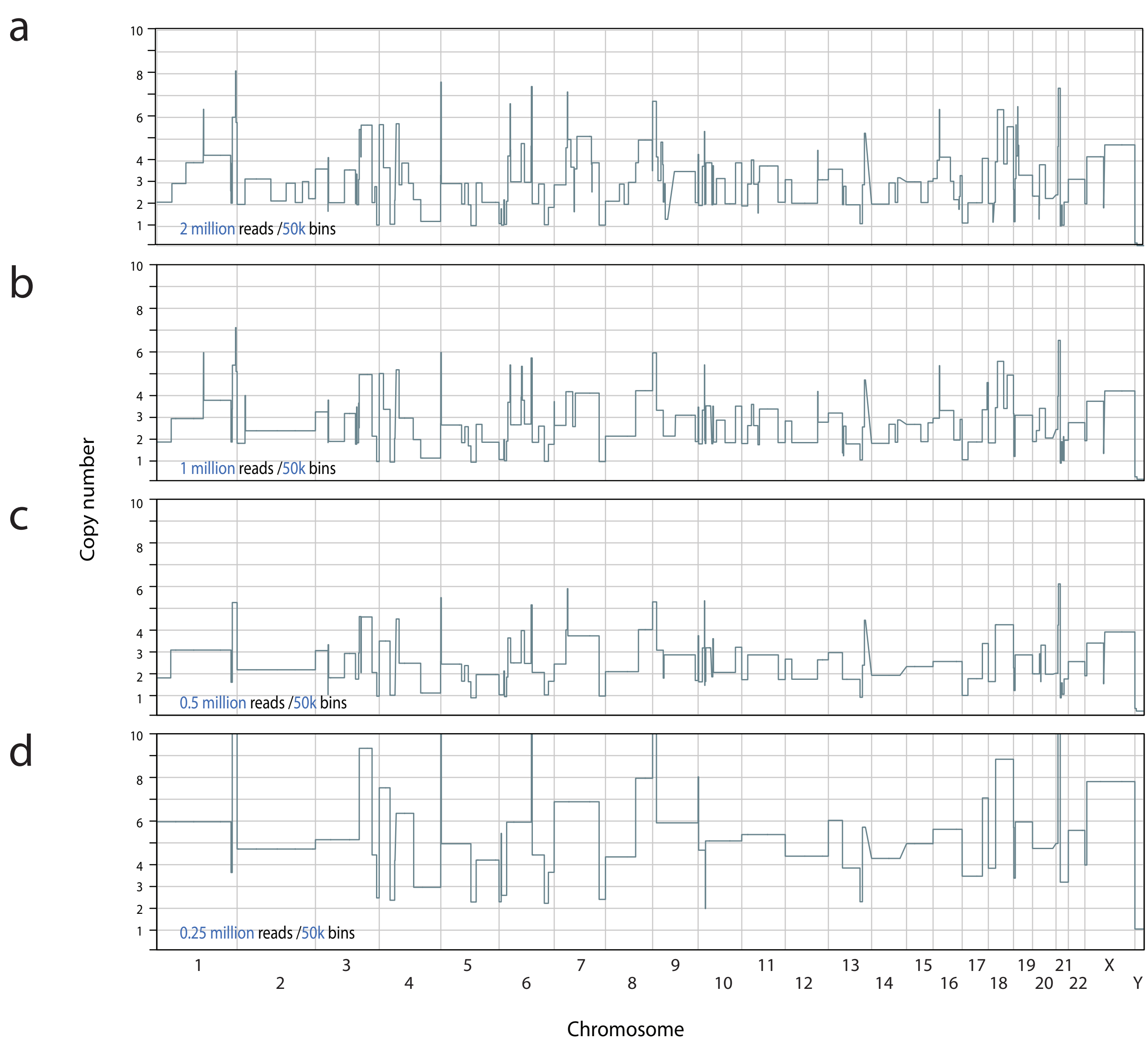

Supplementary Figure 1| Down-sampling beyond 2 million reads using 50K bins results in loss of quantal nature of the CNV data. Genome-wide CNV profiles of rearranged cancer cell using 50K bins with down-sampled data sets at 2 million (a), 1 million (b) , 0.5 million (c), and 0.25 million sequencing reads (d).

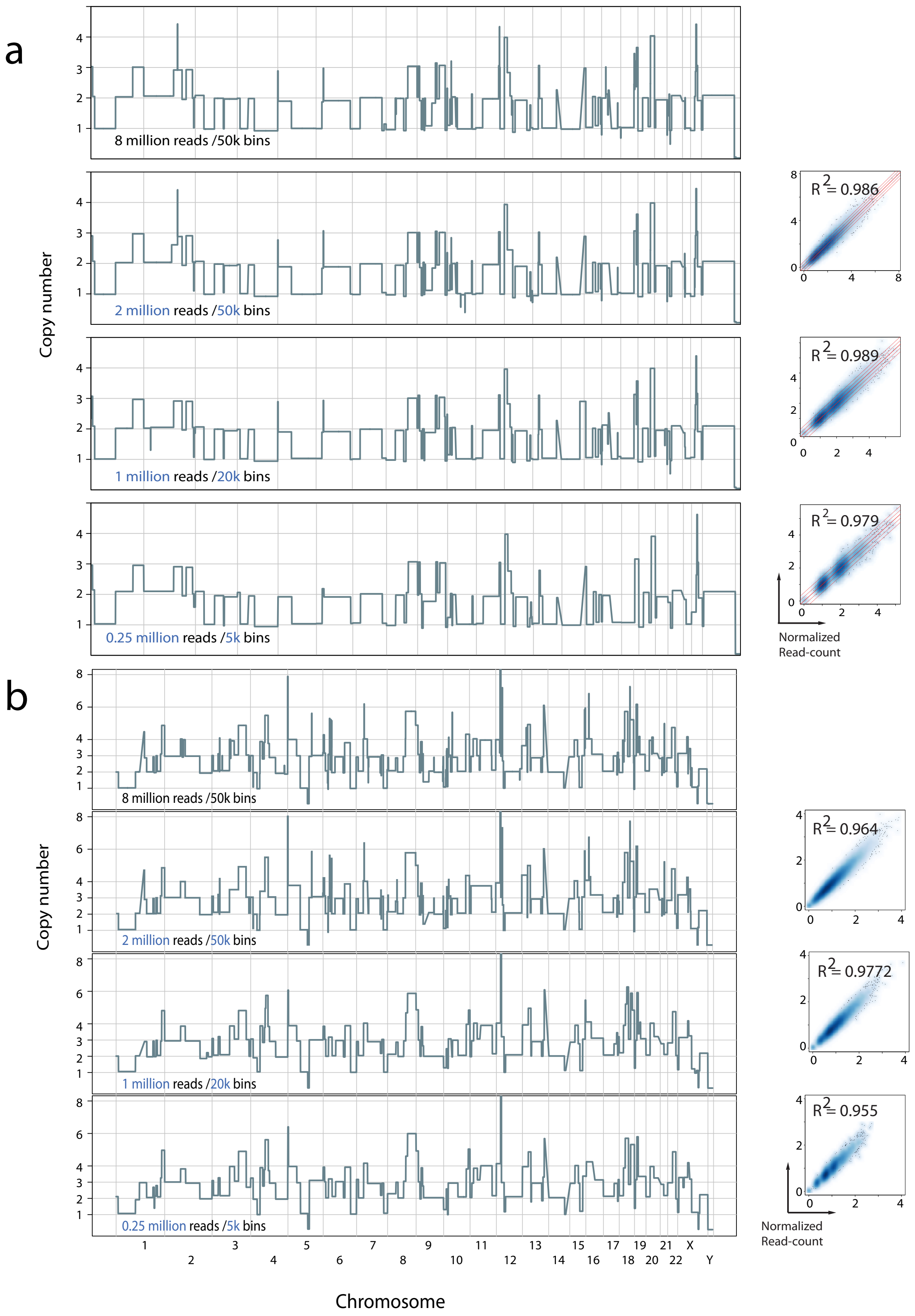

Supplementary Figure 2| Identified minimal read requirement parameters apply to rearranged cancer cells that display varying DNA content.
(a) DNA content=1.6N and (b) DNA content=2.65N. Left panels display genome wide CNV profiles of single cell at different bin resolutions using the original data (8 million reads) or down-sampled data as indicated. Right panels illustrate the correlation values of the down-sampled data sets with the original 8 million read data.

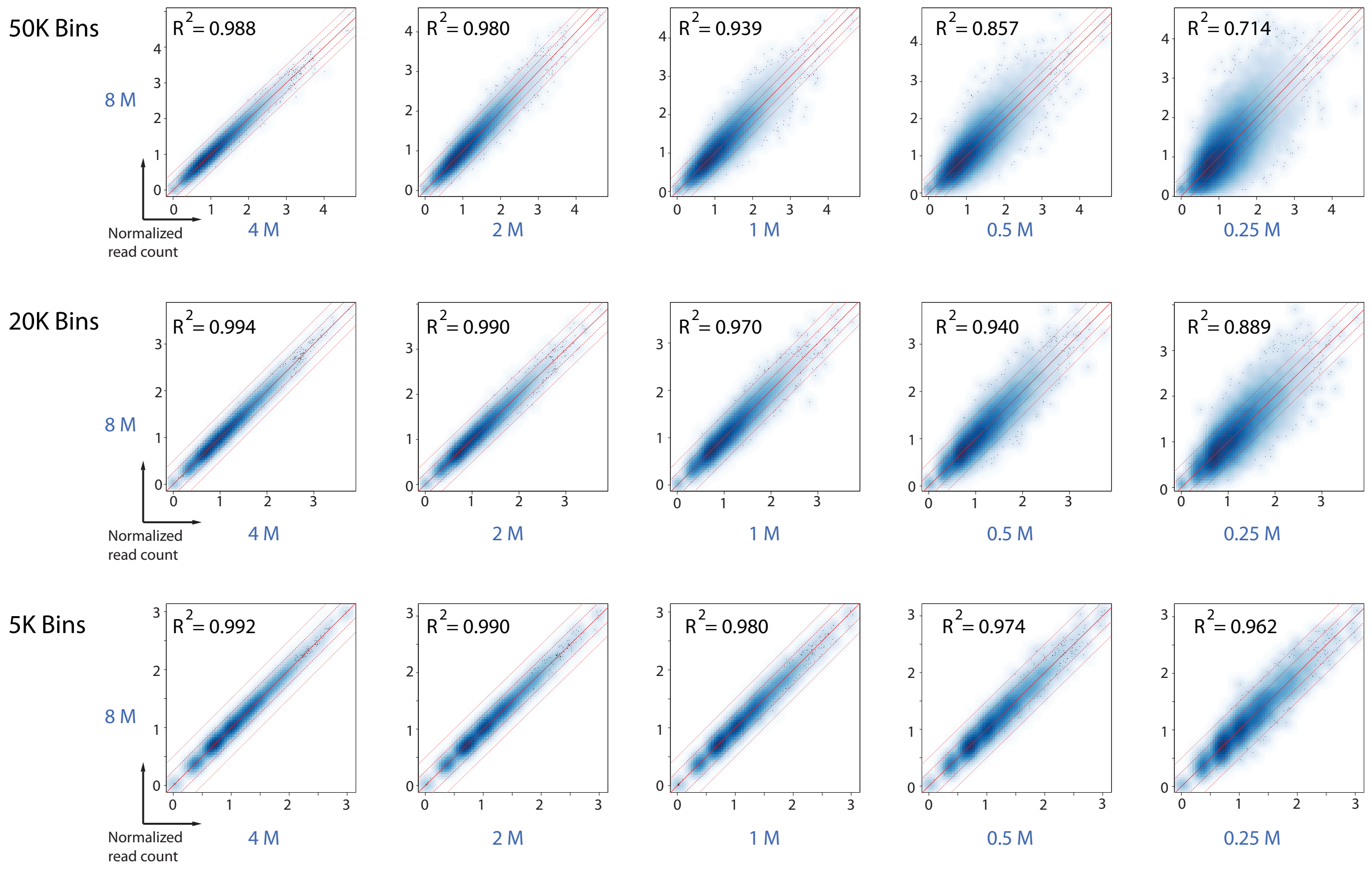

Supplementary Figure 3| Correlation plots of down-sampled data sets from single cell in Figure 1 across different bin lengths. Scatter density correlation plots of normalized read count data of original 8 million read data set with down-sampled data sets. 2,1, and 0.25 million reads at 50K, 20K, and 5K respectively provide strong correlations with original 8 million read data set. Notice increasing correlation with decreased # of bins across down-sampled data. Pearson correlation coefficients of data sets are displayed.

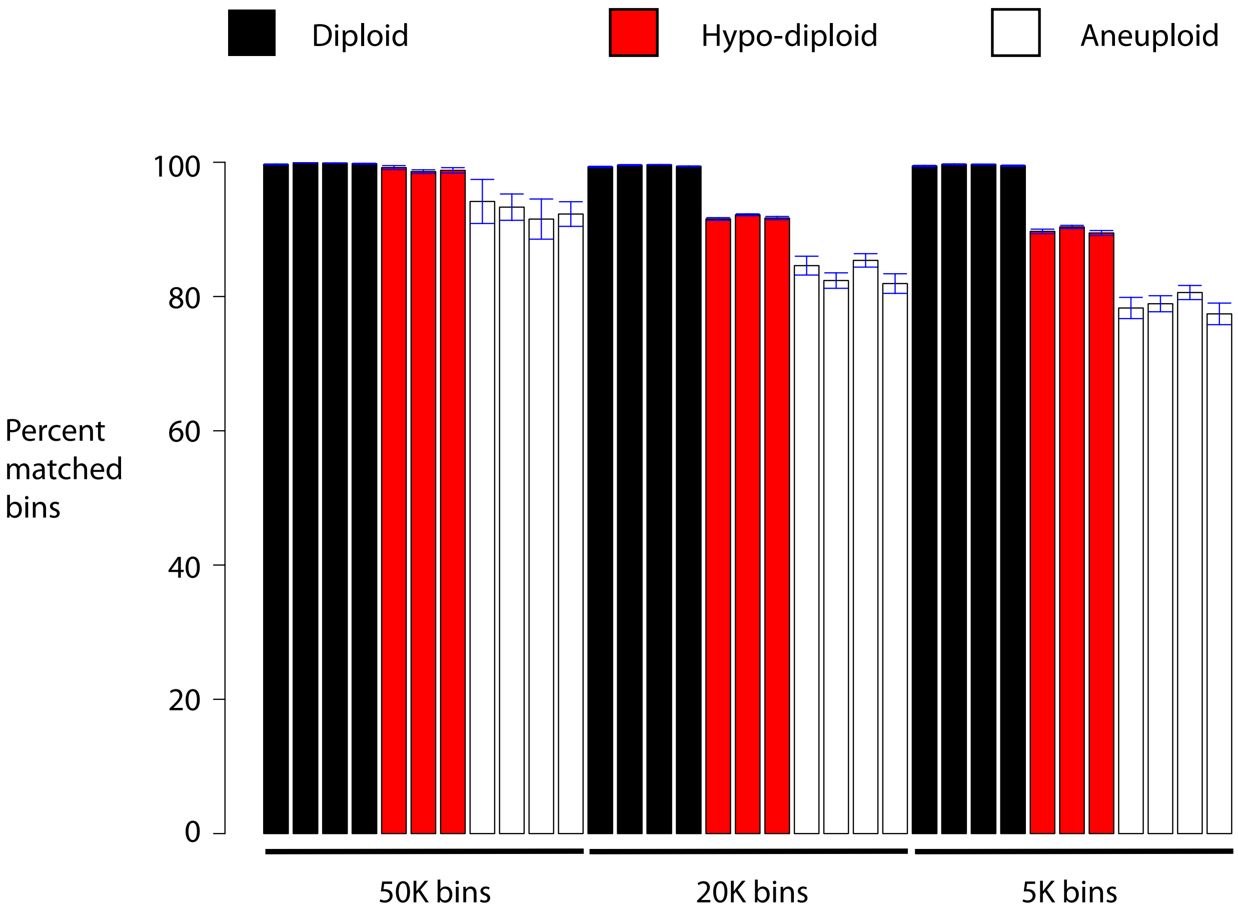

Supplementary Figure 4| Downsampling analysis of copy number variation of single cell genomes of varying ploidy levels at different bin resolutions.
Four diploid, three hypo-diploid, and four aneuploid single cell sequence data sets were downsampled, analyzed at different bin resolutions, and plotted as a function of percent match bins of the original data set. Error bars are in blue. Details are described in the main text and the methods section.

a

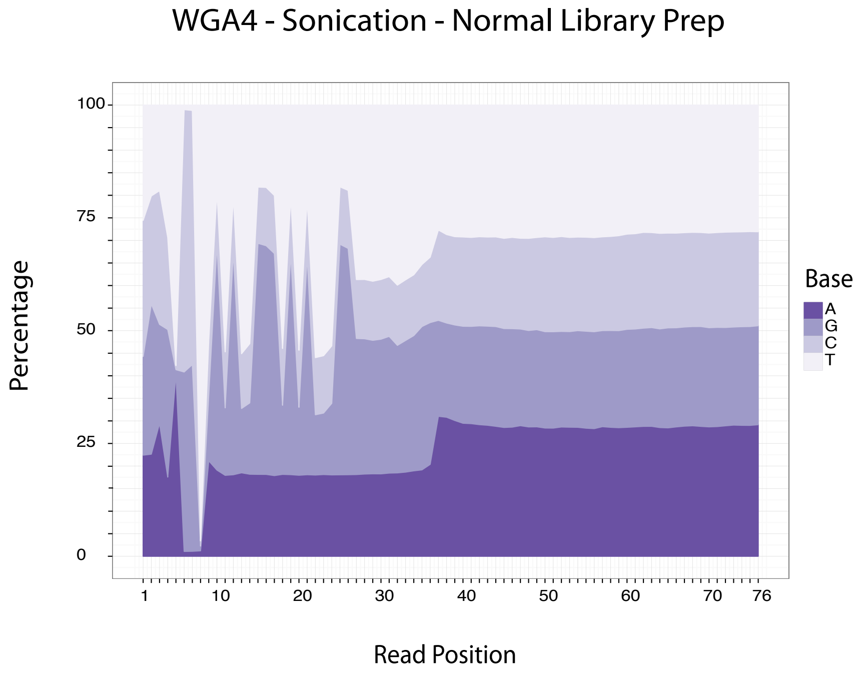

b

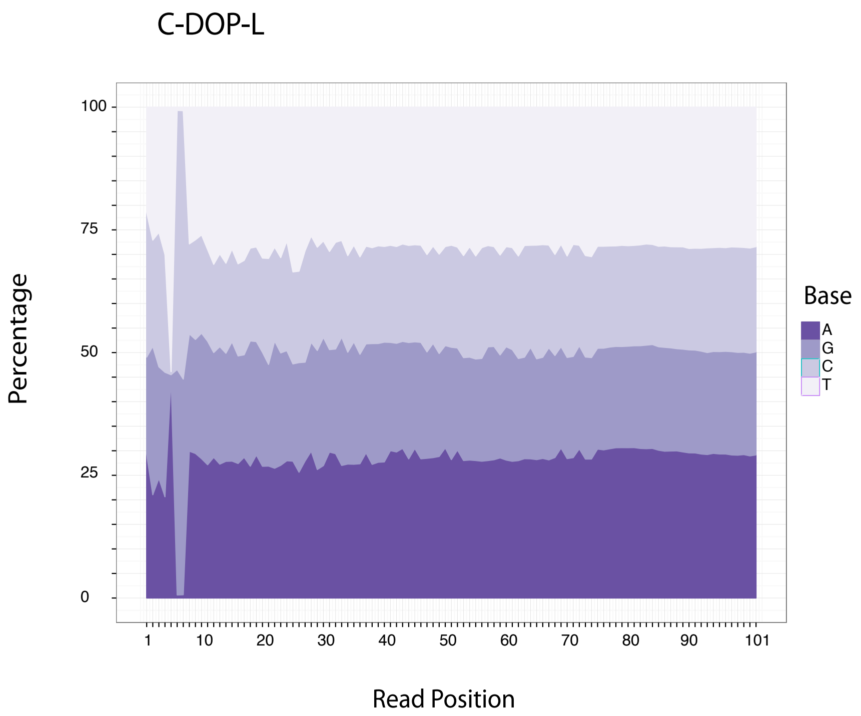

Supplementary Figure 5| C-DOP-L approach removes WGA universal sequences to restored nucleotide diversity in sequenced DNA. (a) Nucleotide distribution plot of WGA DNA processed with DNA sonication and standard Illumina library generation protocols. Notice, uneven nucleotide distributions are observed until read 38. This is due to custom barcodes used (first 8 nucleotides) followed by the 30 base pair universal adaptor sequence, which sonication does not effectively remove. (b) Nucleotide distribution plot of WGA DNA processed using the C-DOP-L method. Notice the restoration of DNA complexity after read 8 (Nucleotides 1-8 are from the custom Illumina barcodes utilized)

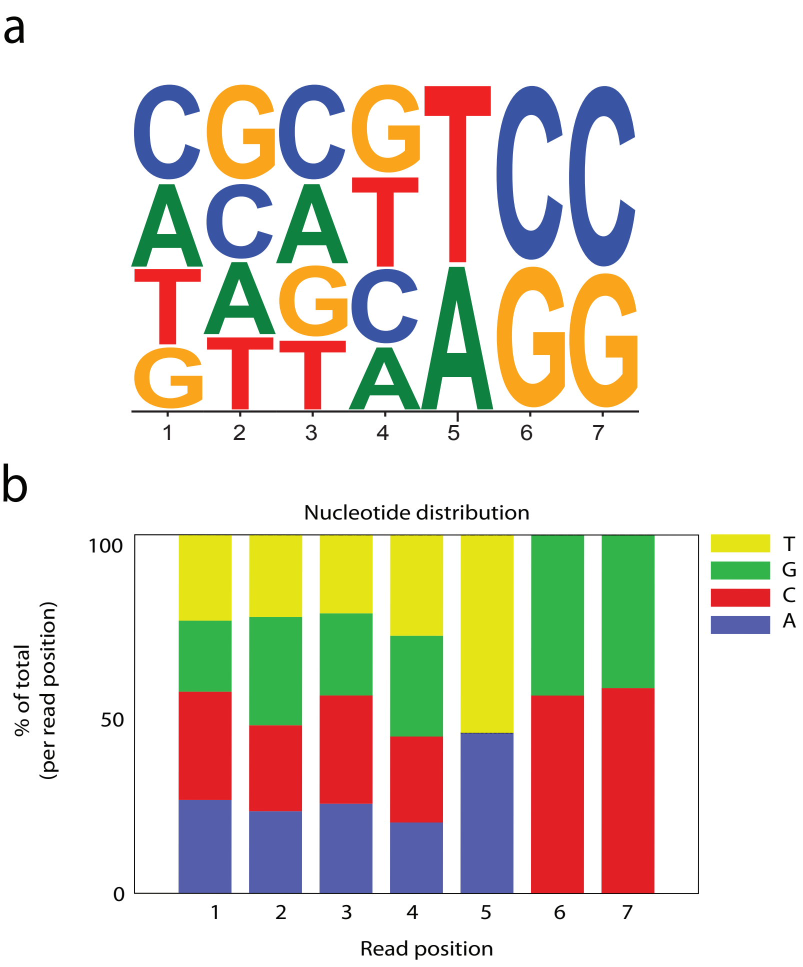

Supplementary Figure 6| Schematic presentation of the sequences of the custom Illumina barcodes utilized in the C-DOP-L method.
(a) Barcode nucleotide distributions of all 96 barcodes plotted in WebLogo format. (b) Chart plot of nucleotide composition of all 96 barcodes utilized.

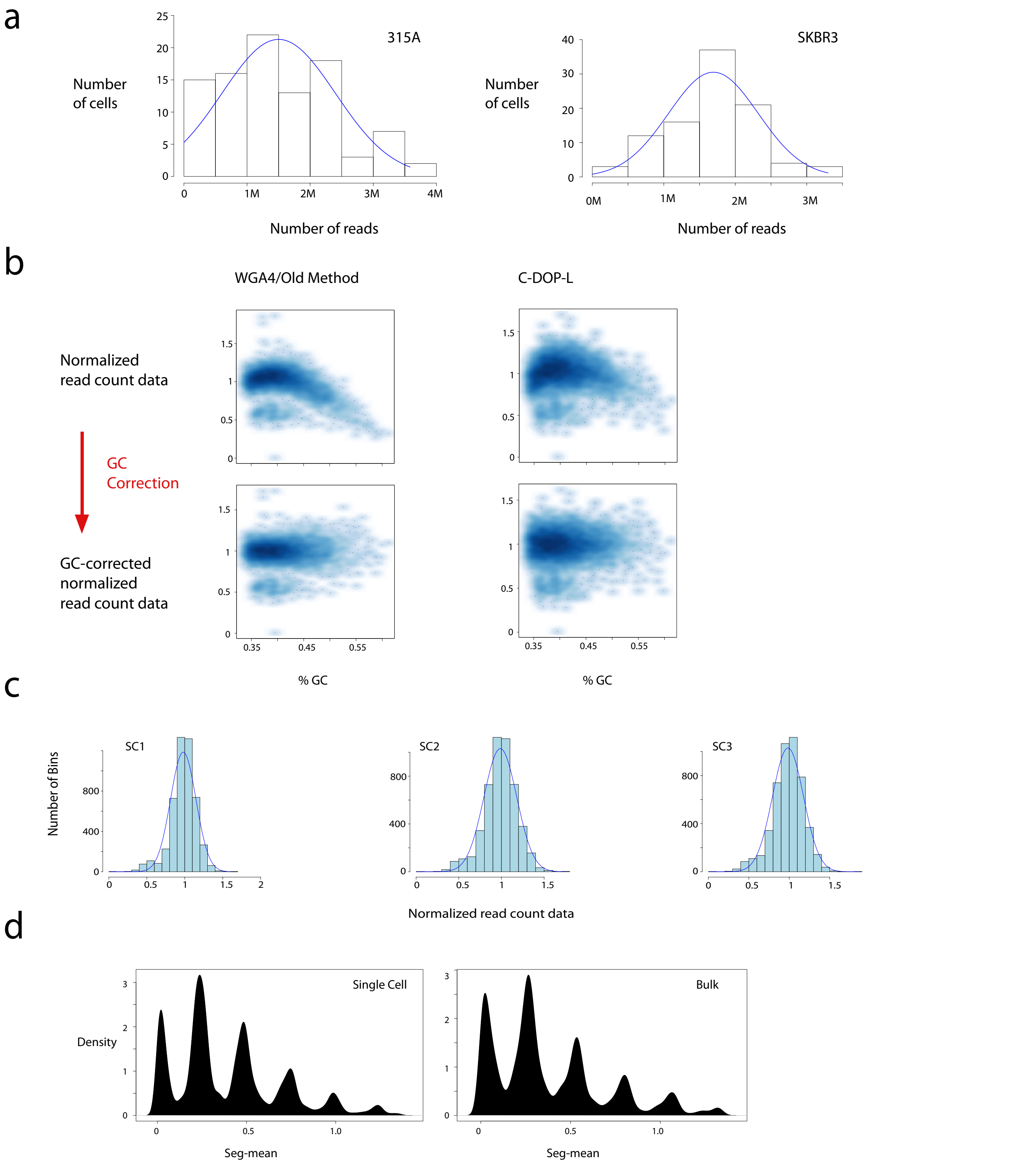

Supplementary Figure 7| C-DOP-L approach facilitates robust determination of high quality CNV data from single cells. (a) Histogram illustration of sequence read counts for all multiplexed single cells libraries from 315 cells (left) and SKBR3 (right). All sequenced cells have a minimum sequence count that is above 0.25 million reads. Average number of sequencing reads per cell was approximately 1.5 million (b) Scatter density plots of the bin count of sequencing reads plotted according to bin GC content. Left panel using the old approach (WGA4) and right panel using the new approach (C-DOP-L). Lowess normalization corrects for the bias. Density plots for GC corrected normalized read counts are displayed. (c) Histogram distributions of normalized read count data of diploid karyotypically normal single cells illustrating uniformity of the whole genome amplification reaction. (d) Smoothened kernel density plots of the pair-wise differences in segmentation values for single as well as bulk SK-BR-3 profiles demonstrating the quantized nature of the data.

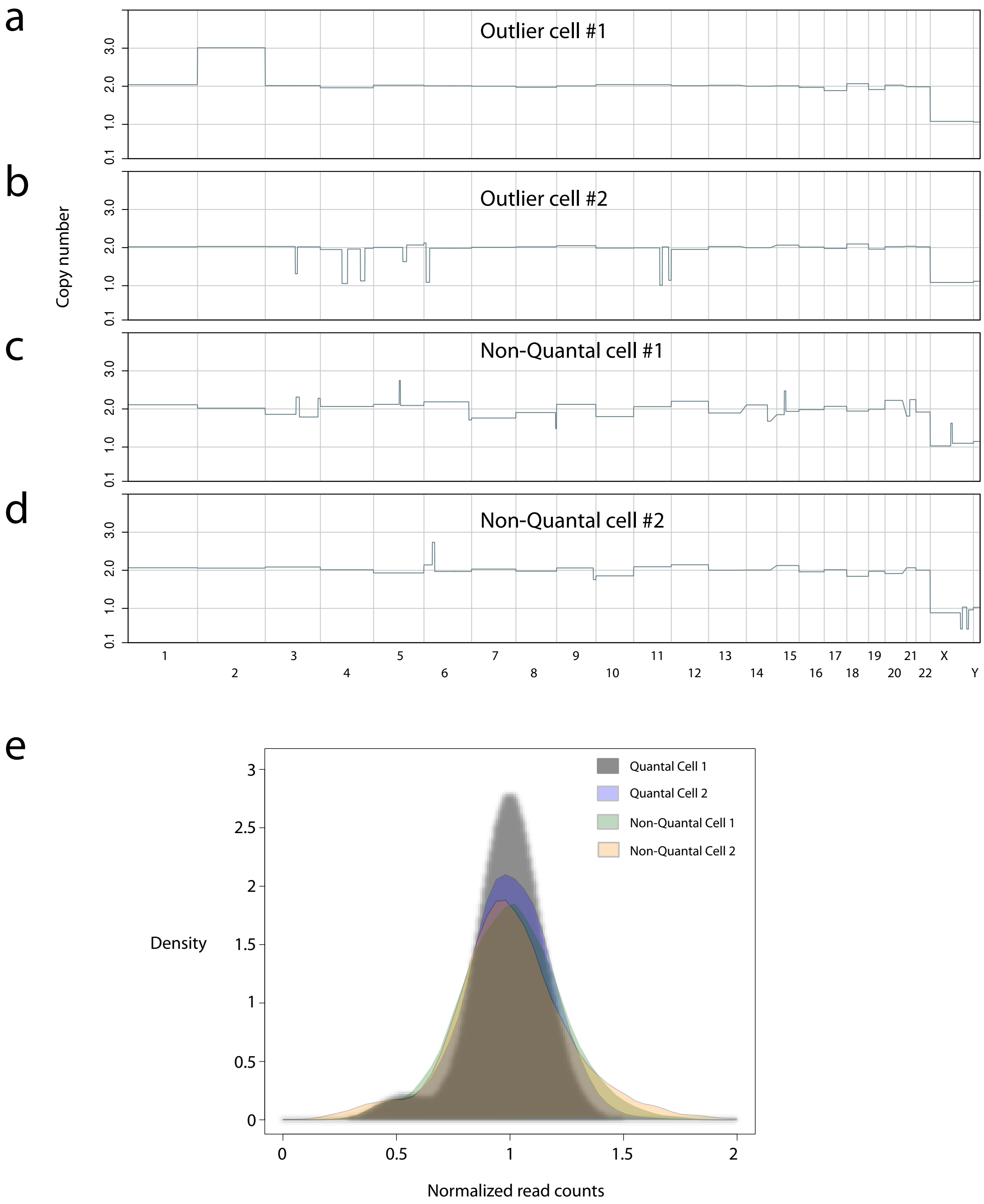

Supplementary Figure 8| Genome alterations identified in normal cells. Representative CNV plots of outlier cells with somatic CNVs (a,b). Representative CNV plots of Non-quantal cells observed (c,d). (e) Smoothened histogram distributions of normalized read count data in bins comparing cells with/without quantized copy number states.

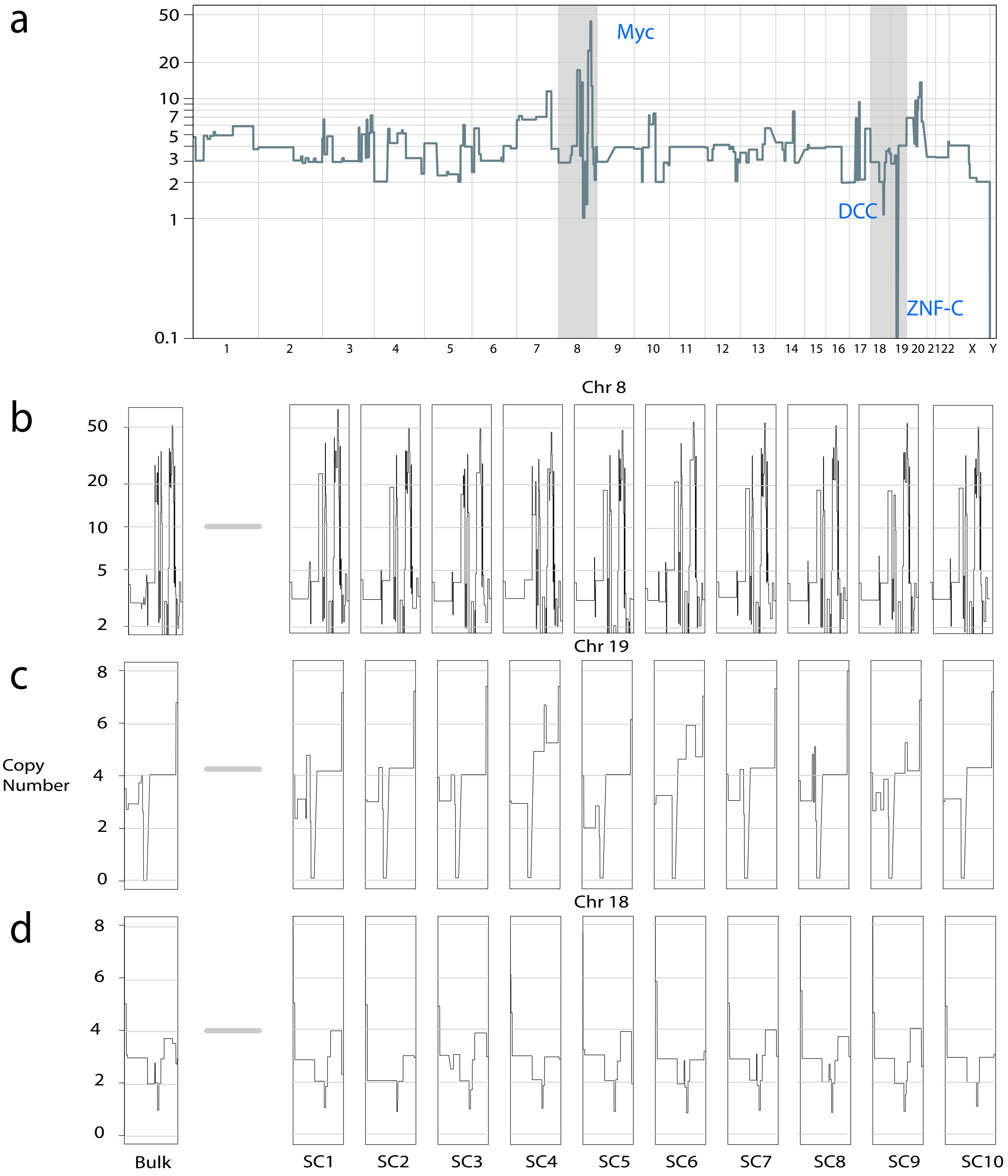

Supplementary Figure 9| Potential "driver" copy number alterations in the SK-BR-3 genome are identified in 100% of single cancer cells sequenced. (a) Representative view of a bulk SK-BR-3 genome. Gray shaded rectangle highlight chromosomes containing potential driver alterations. (b,c,d) Comparative zoom-in views of bulk and 10 random single cells of chromosomes containing amplifications (b), homozygous deletions (c), and heterozygous deletions (d). SC refers to single cell. Myc, CMYC. DCC, netrin 1 receptor. ZNF-C, Zinc finger cluster.

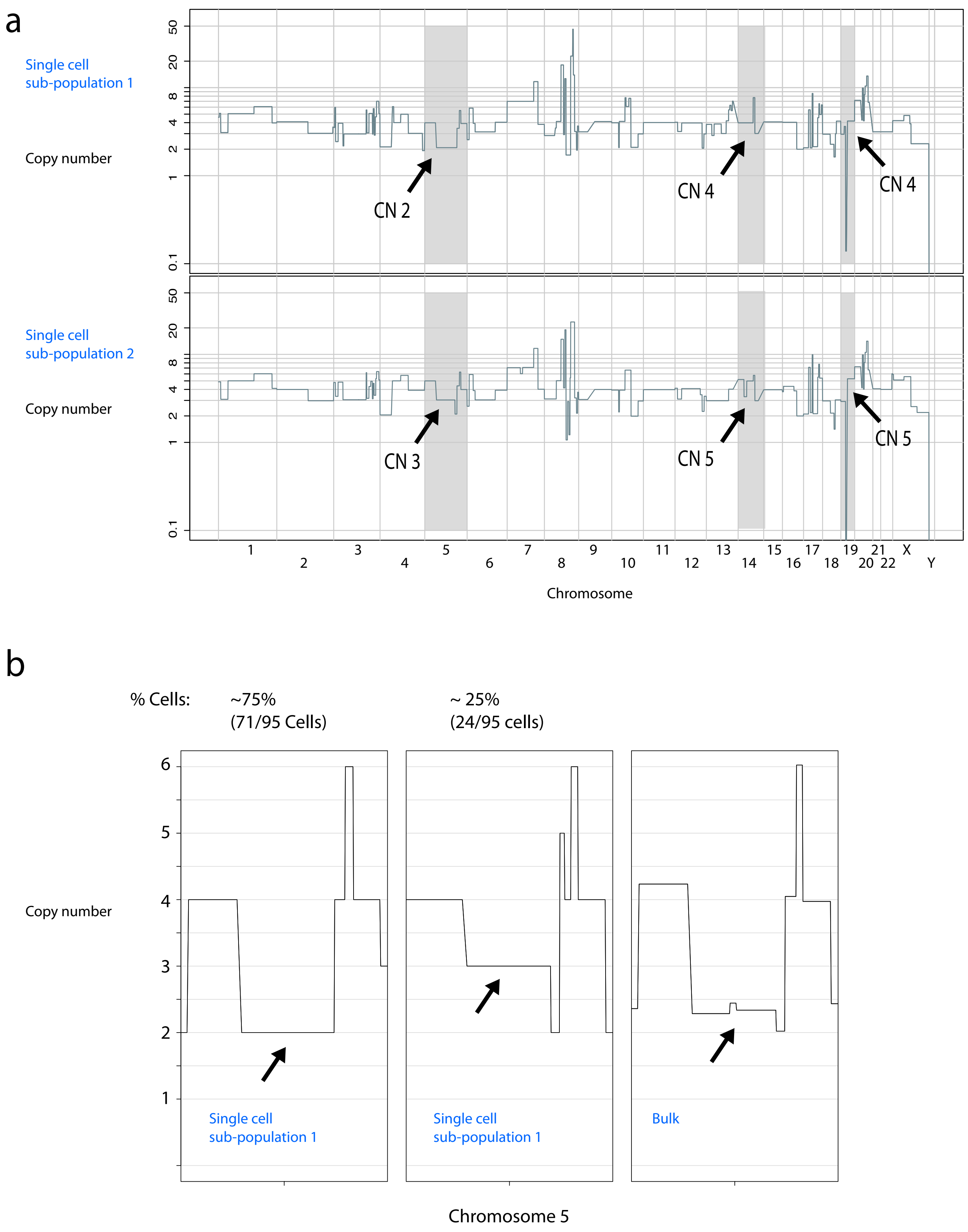

Supplementary Figure 10| Copy number variation between cells of the different SK-BR-3 sub-populations. (a) Genome wide CNV plots of a representative single cell from sub-population 1 (Top Panel) and sub-population 2 (Lower Panel). Example chromosomes where copy number states differ between different single cells are shaded in gray. Black arrows point to the regions on the chromosomes where the CNVs occur. (b) Example of sub-clonal variation identified via single-cell sequencing that is also evident in shot-gun bulk deep whole genome sequencing.

a

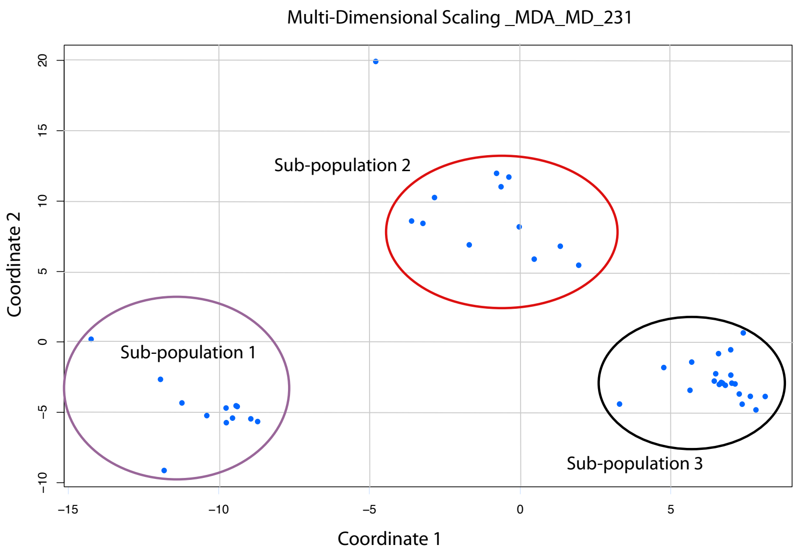

b

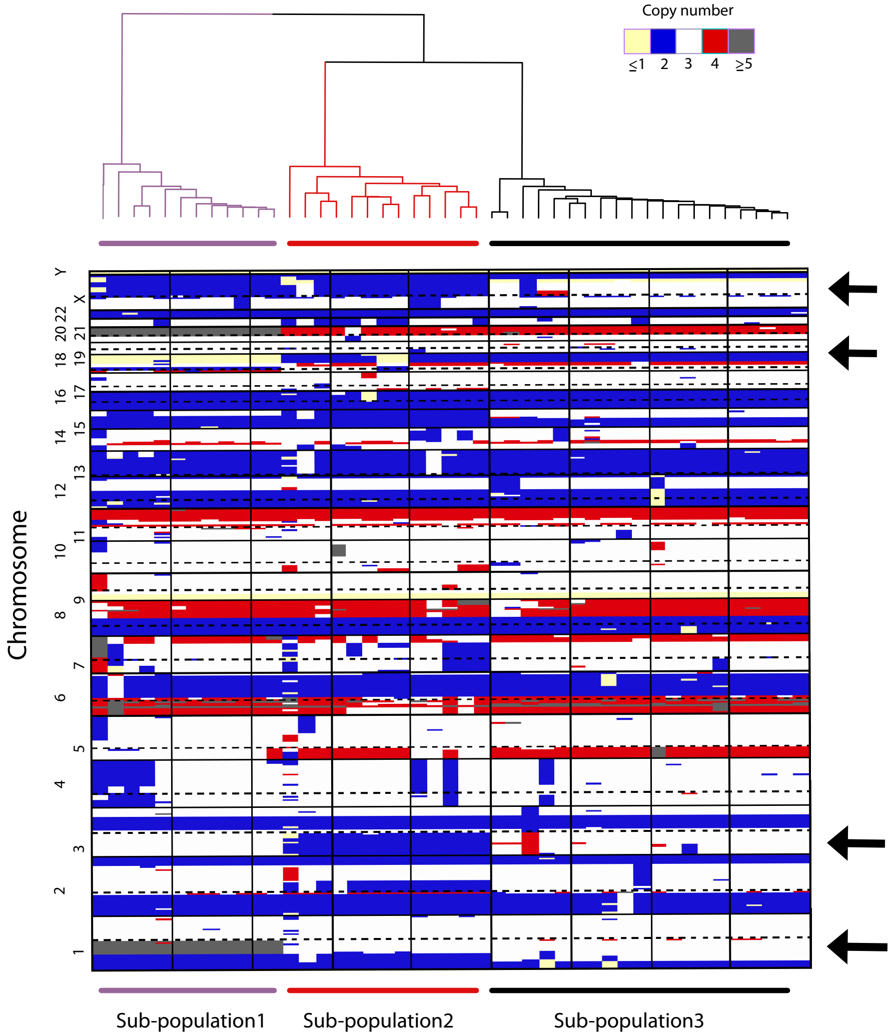

Supplementary Figure 11| Intra-tumoral heterogeneity and sub-clonal populations in the MDA-MB-231 breast cancer cell line. (a) Multi-dimensional scaling of 45 single cell genomes. (b) Hierarchical clustering heatmap of single cell genomes. Black arrows point to genomic regions that are differ between the 3 different sub-populations.

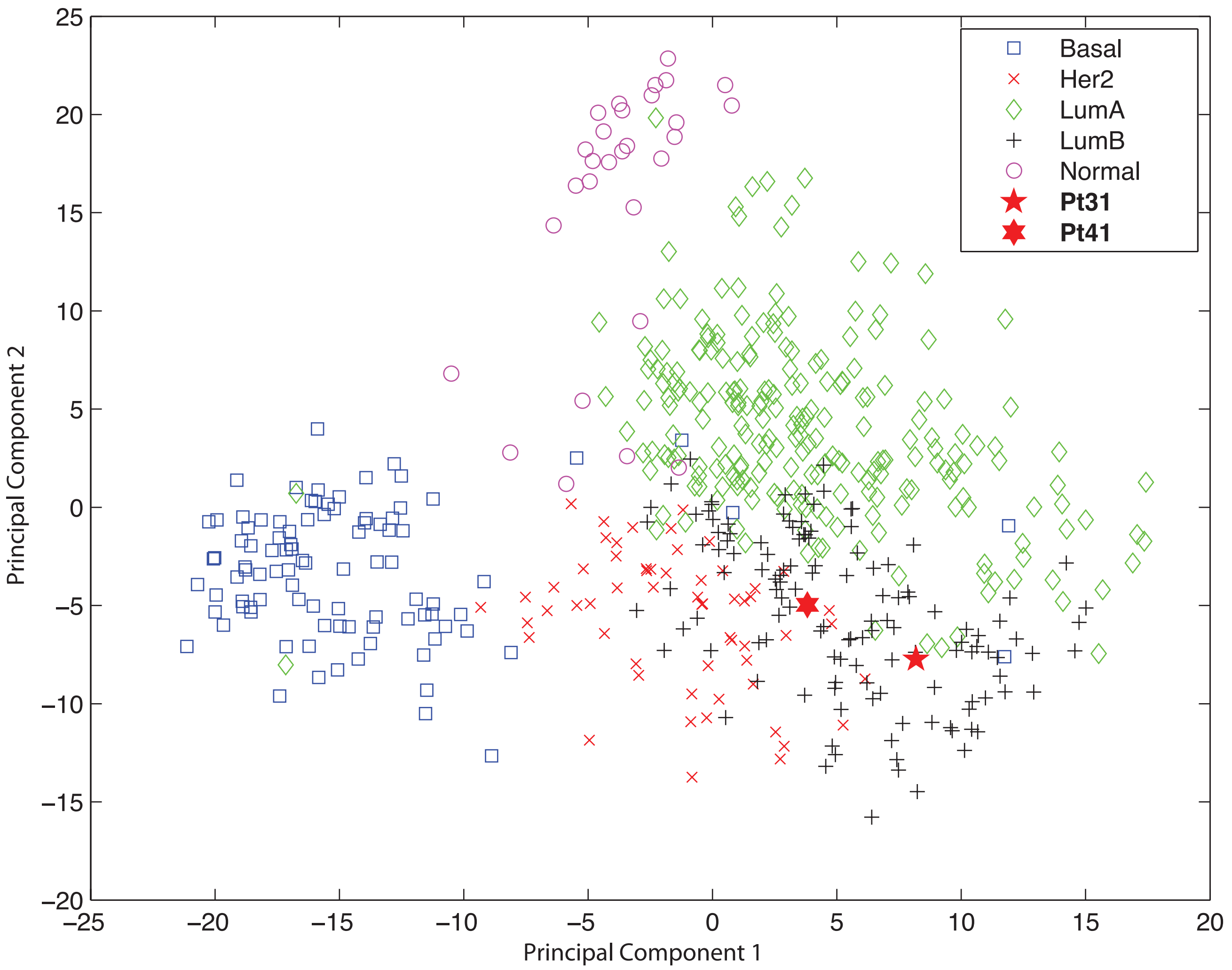

Supplementary Figure 12| Pt41 and Pt31 belong to the Luminal B breast cancer gene expression subgroup. 542 TCGA samples were projected on the PCA plot with the top 2 principal components and given subtype information obtained from TCGA data sets (see methods). It included 96, 58, 231, 128 and 29 samples respectively for Basal, Her2, LumA, LumB and Normal-like subtypes. Pt31 and Pt41 were projected on the background within the LumB cluster marked as a pentagram and a hexagon.

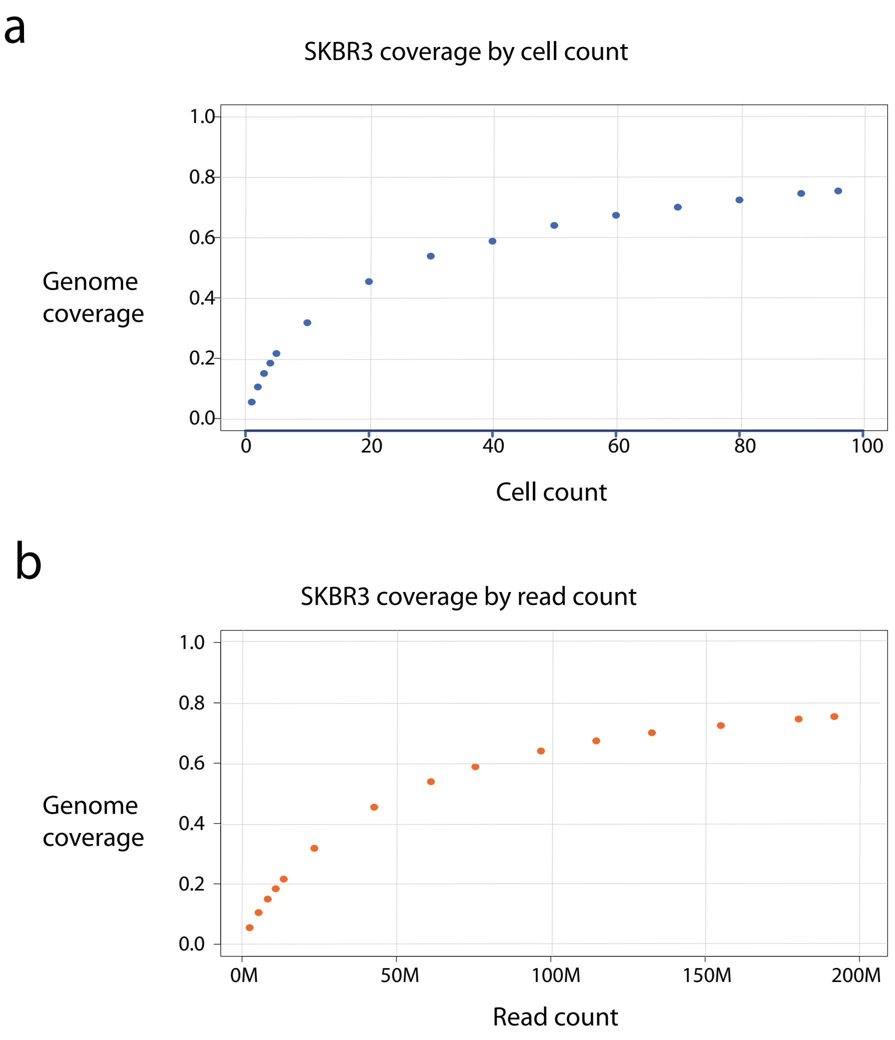

Supplementary Figure 13| Genome coverage increases with more single cells sequenced. Fraction of genome covered at a minimun of 1X with (a) increasing numbers of single cells sequenced and (b) increasing number of sequencing reads

Step	Cost ($ Per Cell)	Time
Single Cell Amplification (**SEQXE)**	15.24	~ 5 hrs
WGA DNA Purification (**QIAQuick 96 PCR Purification Kit)**	1.3	~ 1 hrs
Digestion/Purification of WGA DNA **(SEQXE + Agencourt AMPure Beads)**	1.14	~ 4 hrs
Ligation of Illumina Barcoded Adaptors +Amplification **(Quick Ligation Kit + Phusion polymerase)**	2.6	~ 1 hrs
Illumina 76 Single Read Sequencing **(HiSeq Machine)**	11.62	~ 5 days
C-DOP-L Procedure to Data	31.9	~ 12 hrs (to Sequence Ready Libraries)

Supplementary Table 1: Cost and time analysis for each step of the C-DOP-L method
